# Advanced Glycerol Oxidation to Formic Acid in a Multiphasic Jet Loop Reactor Using Polyoxometalate Catalysts

**DOI:** 10.1021/acssuschemeng.5c10177

**Published:** 2025-12-17

**Authors:** Ira Christina Wirth, Daniel Niehaus, Dorothea Voß, Michael Schlüter, Jakob Albert

**Affiliations:** 1 Institute of Technical and Macromolecular Chemistry, 14915 Universität Hamburg, Bundesstrasse 45, Hamburg 20146, Germany; 2 Institute of Multiphase Flows, Technische Universität Hamburg, Eißendorfer Strasse 38, Hamburg 21073, Germany

**Keywords:** jet loop reactor, process intensification, glycerol oxidation, formic acid, polyoxometalate catalyst

## Abstract

Glycerol is a common byproduct of commercial biodiesel production and can be used for the production of green platform chemicals such as biogenic formic acid (FA). Biogenic FA is industrially produced via selective catalytic oxidation in the OxFA process using various biomass in conventional stirred-tank reactors (STR). However, the reaction is limited by the low oxygen solubility in the aqueous reaction media that typically requires high oxygen pressures of 10–30 bar. This study aims to implement the multiphasic selective oxidation of glycerol to FA in a jet loop reactor (JLR), highlighting the economic and mass transfer advantages compared to the conventional used STR. The multiphasic approach was catalyzed by the homogeneous H_5_PV_2_Mo_10_O_40_ (HPA-2) polyoxometalate catalyst, already established in the commercial OxFA process. The reactor characterization indicates an efficient and high gas–liquid mass transfer, achieving volumetric mass transfer coefficient values (*k*_l_ · *a* values) ranging from 51 to 173 h^–1^. Afterward, the multiphasic glycerol oxidation reaction to FA was implemented and compared in both reactor concepts under identical reaction conditions. The JLR achieved very high FA space-time-yields (STY) of up to 30.0 g_FA_ L_R_^–1^ h^–1^, highlighting its improved mass transfer and favorable economics already at 5 bar oxygen pressure in a simple glass setup. Determination of the kinetic parameters in the JLR resulted in reaction orders of 0.83 for glycerol and 0.54 for oxygen underlining the importance of an efficient gas–liquid mass transfer. Moreover, the activation energy was determined to be 78.3 kJ mol^–1^, which is well in line with previous studies carried out for the OxFA process in a STR. The calculated Hatta number of 0.014 for the multiphasic glycerol oxidation in the JLR indicates that the reaction is in the kinetic regime already at low oxygen pressures of 5 bar demonstrating the high potential of the reactor concept for future studies.

## Graphical abstract

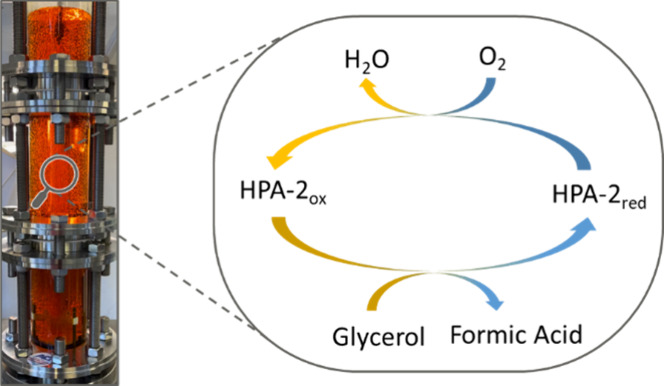

## Introduction

The increase in greenhouse gas emissions due to human activities, primarily from burning fossil fuels, amplifies the greenhouse effect and leads to global warming.^1^ These changes contribute to extreme weather, which have significant ecological and societal impacts and pose a global threat.^2,3^ Due to these circumstances and the fact that the production of most platform chemicals is still based on fossil resources, there is a growing interest in the sustainable production of platform chemicals.^4^ Biodiesel synthesis could be a sustainable alternative to fossil diesel production. Biodiesel is a mix of fatty acid methyl esters, which are obtained through the transesterification of fats and oils. Herein, glycerol is obtained as a byproduct.^5^ Glycerol is currently used in pharmaceuticals, cosmetics, e-cigarettes and the food industry.^6^ However, the increase in biodiesel production is flooding the market with glycerol. As a result, there is a growing interest in utilizing glycerol to ensure the competitiveness of biodiesel production.^6^

One approach is to utilize glycerol by catalytic oxidation to produce C_1_–C_3_ oxidation products. The reviews by Katryniok et al.^7^ and Hu et al.^8^ summarize the state-of-the-art in catalytic glycerol oxidation. So far, several approaches for the oxidation of glycerol have been followed using electro-, photo-, and thermal oxidation pathways to produce high value C_2_ and C_3_ products.^9–11^ In addition to C_2_ and C_3_ pathways, glycerol oxidation to C_1_ products can yield in the important C_1_ intermediate formic acid (FA). FA is a bulk chemical that is currently produced mainly from fossil fuels using carbonylation of methanol.^12^ Finding a way to obtain biogenic FA is a main focus of current research and opens a way of using FA as a green feedstock for replacing fossil resources.^13^

An innovative, sustainable, and commercialized method to obtain FA from biomass is the OxFA process.^14,15^ Herein, biogenic FA can be produced from a diverse range of biomass under mild reaction conditions.^16,17^ FA is currently used in the textile^18^ and rubber industries.^19^ FA could be of interest in the future as a promising hydrogen storage medium^20–22^ and as a liquid synthesis gas equivalent.^23,24^ The oxidation reaction in the OxFA process is homogeneously catalyzed by polyoxometalates (POMs).^25^ POMs are complex inorganic metal oxide compounds consisting of oxygen and light transition metals preferably in their highest oxidation state.^26,27^ If heteroatoms are incorporated into the POM structure, they are referred to as heteropolyanions (HPAs).^28^ When protonated, HPAs have a high Bro̷nsted acidity^29^ and catalyze several redox reactions under mild conditions.^30–32^ The OxFA process applies vanadium-substituted phosphomolybdate-HPAs as catalyst, molecular oxygen as oxidant, and water as environmentally benign solvent.^33^ Usually, the reaction is carried out at pressures below 30 bar and temperatures below 120 °C. The HPA catalyst oxidizes the biomass and thereby gets reduced changing its oxidation state from V^5+^ to V^4+^ in solution. Consecutively, oxygen reoxidizes it back to V^5+^ and thereby closes the catalytic cycle.^14,34,35^ The mass transfer of oxygen into the liquid phase is crucial for the reoxidation of the catalyst. The review by Katryniok et al.^7^ mentions the great importance of overcoming the mass transfer resistance and entering the chemical limiting regime. Herein, the kinetics of the reaction can be determined and the reaction is more controllable. Mass transfer can be optimized by changing the reaction conditions (e.g., increasing pressure, stirrer speed) and by the choice of reactor. The stirred tank reactor (STR) has traditionally being used for biomass oxidation reactions like the OxFA process and glycerol oxidation.^7,36,37^ One of the main disadvantages of STRs unlike other reactor concepts, is their loss in efficiency at large scale (from 200 m^3^) due to mechanically limited volumetric power input.^38^ To counteract this effect, other reactor concepts such as bubble columns are used, which on the other hand are limited by volumetric power inputs.^39^ Alternative reactor concepts for glucose oxidation to FA have already been introduced by Ponce et al.^40,41^ and Wei et al.^42,43^ Ponce et al.^44^ performed glucose oxidation to FA in a liquid core waveguide membrane microreactor with high gas-to-liquid mass transfer. Wei et al.^42,43^ integrated glucose oxidation to FA in microreactors with Taylor flow. In Taylor flow, gas bubbles and liquid slugs alternate in the microchannel, creating a high interface and favoring gas to liquid mass transfer. Moreover, Krueger et al.^45^ optimized this concept also for xylose oxidation and the commercial C5-hydrolysate Renmatix from the Plantrose process.

Besides the advantages of the previous reactor types for selective biomass oxidation, the Jet-loop reactor (JLR) is a reactor concept that also favors mass transfer in biphasic systems by generating high surface areas with low energy consumption and offering the possibility of easy scale-up.^46^ In general, a JLR consists of a cylindrical body with a draft tube in the center separating the downstream section from the upstream section. The jet nozzle is located at the center above the draft tube. JLRs operate with an inner and an outer recirculation flow. The external recirculation flow is generated by circulating the medium through a pump (schematic representation in Figure 1).

**Figure 1:**
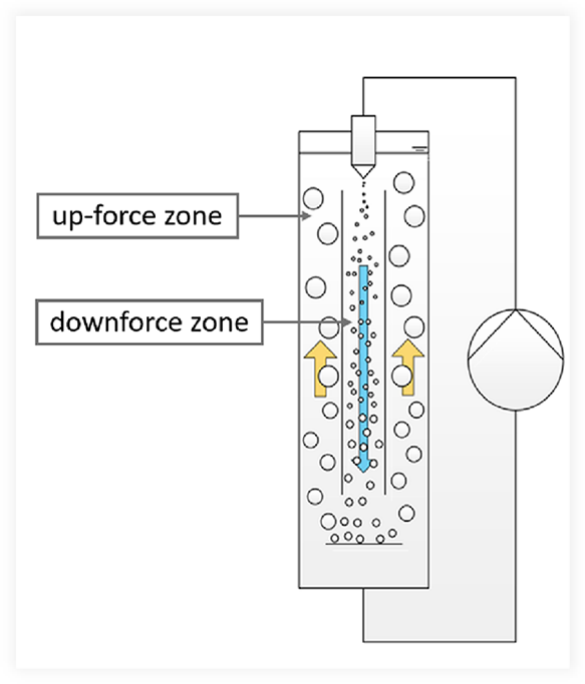
Schematic representation of a gas/liquid jet loop reactor with illustration of the flow directions based on the illustration of Warmeling et al.^46^

The JLR is characterized by its excellent mixing performance and the fact that there are no moving parts in the reactor center, which makes it much easier to seal the reactor and to conduct high pressure experiments.^47^

The generation of high surface areas and the excellent mixing performance produce a fine dispersion of reactants and promotes evenly distributed heat transfer, reducing hotspots in the reactor and minimizing the risk of a thermal runaway. High interfacial areas and uniformly distributed heat transfer is advantageous in terms of reaction performance and safety.

A few two-phase reactions have already been implemented in JLRs and intensely invested. For example, Warmeling et al.^48^ already carried out hydroformylation in a JLR and Roth et al.^49^ optimized the aqueous biphasic hydroformylation of oleochemicals in a JLR. Another application of a JLR is its use in phenol removal by ozonolysis, as demonstrated by Barlak et al.^50^These reactions benefit from an increase in specific surface area, which can be controlled through nozzle design and recirculation velocity. Specific surface area is one of the primary factors that positively influence mass transfer in multiphasic reactions. Despite this, to our knowledge, the use of JLRs for homogeneously catalyzed reactions, in particular oxidation reactions, has only hardly been investigated.

The aim of this study is to perform the selective catalytic oxidation of glycerol to FA analogous to the OxFA process in a JLR setup with in situ analytics to overcome the so-far limiting mass transfer of oxygen into the aqueous reaction medium in conventional STRs and to intensify the process with respect to lower energy input and increased reaction rates at mild reaction conditions. Moreover, the limitations of plug-flow systems like the microreactors described above with respect to solid biomass feedstocks should be resolved.

## Experimental Section

### Setup of the Jet Loop Reactor

The flow sheet for the self-constructed JLR setup for glycerol oxidation is shown in Figure 2. The JLR is constructed with a cylindrical body comprised of three sight glasses (nominal volume 2.44 L, working volume 2.40 and 1.96 L liquid volume) and a central glass draft tube, separating the downstream from the upstream section. A two-substance jet stream nozzle is positioned above the draft tube where both gases (oxygen or nitrogen, V-1 to V-3) and liquid (V-12) can be inserted into the reactor. The gas phase is continuously supplied through the capillary of the nozzle at varying flow rates, controlled by a Bronkhorst Mass Flow Controller (type EL-FLOW Select) for oxygen. The nozzle creates a jet that pushes the liquid into the draft tube and disperses the gas into small bubbles. The gas–liquid mixture is flowing down the draft tube and reflected at the impact plate at the bottom. After flowing up again in the upstream section, the gas–liquid mixture is sucked again by the nozzle and the loop is closed. The catalyst solution can be introduced into the JLR through a ball valve (V-5) using a syringe with a cannula. External recirculation flow is generated by a Scherzinger Pump Technology 4030 circulating the reaction medium. The medium is initially pumped out of the reactor outlet through a PreSens oxygen probe for determining the dissolved oxygen, a heat exchanger from Hebmüller Group to adjust the desired reaction temperature, and directed to the two-substance jet nozzle, from which it returns to the reactor. Liquid samples can be taken from the reactor outlet via a ball valve (V-4).

**Figure 2:**
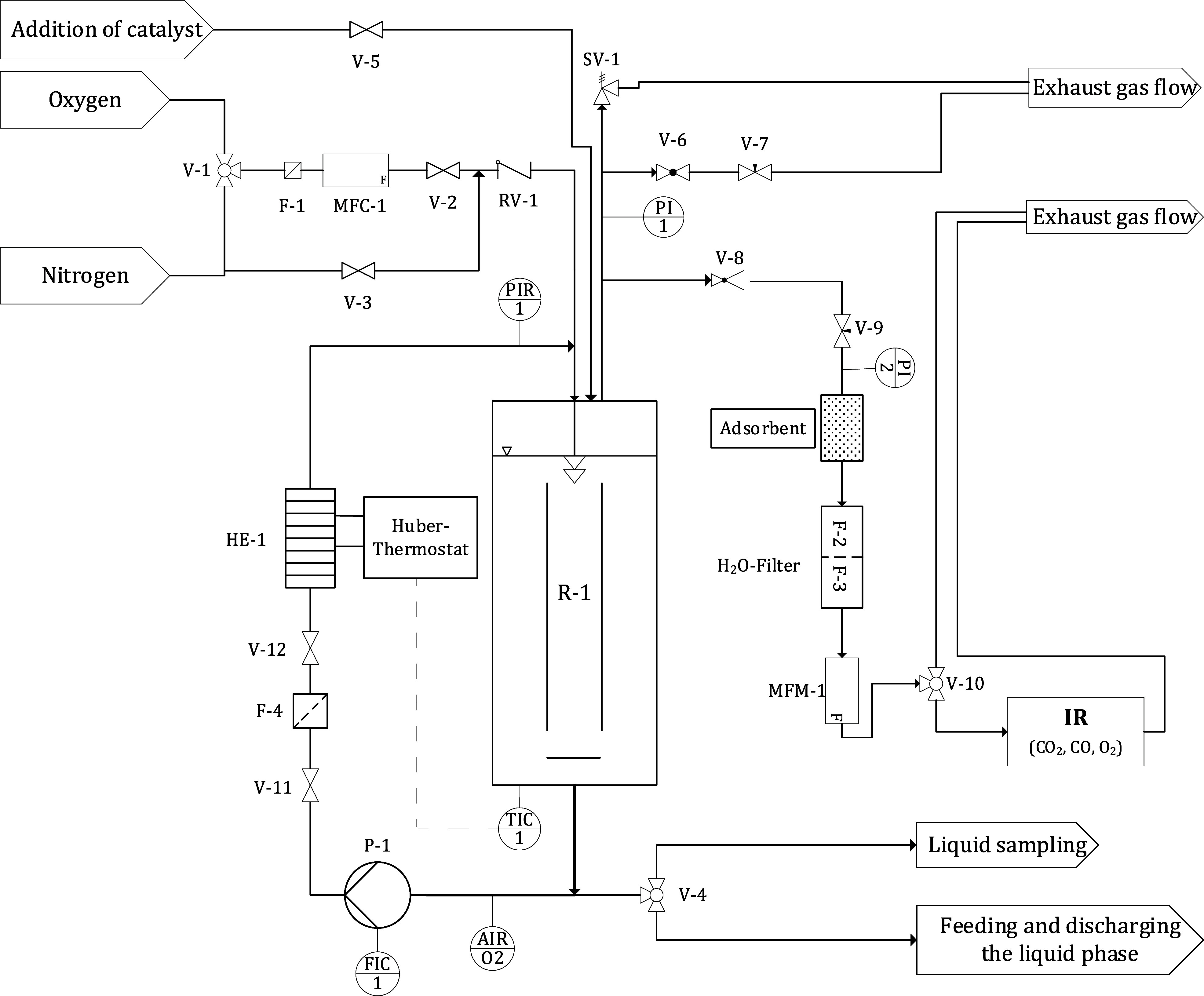
P&I diagram of the jet loop reactor (JLR).

To monitor and analyze the exhaust gas flow, it is discharged in a controlled manner through a ball valve (V-8), followed by a needle valve (V-9) and a pressure gauge (PI 1). For precise measurement of the flue gas flow, a Bronkhorst mass flow meter (MFM) type EL-Flow Prestige is installed. The gas flow is directed from the MFM into an infrared spectrometer (X-STREAM XE) from Emerson Process Management GmbH & Co. OHG for analyzing the exhaust gas composition. This device integrates an infrared sensor for CO and CO_2_ concentration and a paramagnetic oxygen sensor for O_2_ concentration.

### Catalyst Synthesis and Characterization

The synthesis of the HPA-2 (H_5_PV_2_Mo_10_O_40_) catalyst was carried out according to a synthesis protocol of Odyakov et al.^51^ modified by Albert et al.^52^ The detailed synthesis procedure as well as the characterization data are described in the Supporting Information in Section 1.2 and 2 in the Tables S2–S3 and Figures S1–S10.

### Determination of Hydrodynamics and Oxygen Solubility in the JLR

After setting up the JLR, the respective fluid mechanical parameters were determined. First, the liquid volume of the JLR was dogged at different filling levels adjusted by various pump flow rates (Figure S11). The obtained values were plotted and linearized (Figure S12). For the experiments the reactor was filled with water or 20 wt % aqueous glycerol solution and the filling level was recorded to establish the liquid volume from the linear equation. The liquid flow inside the JLR was adjusted using the pump P-1. The gas flow into the JLR was set using MFC-1 with nitrogen, ensuring that the flow rate into the reactor matched the gas flow rate out of the reactor at the MFM-1. For the experiments at room temperature, the nitrogen was then displaced by oxygen. For the experiments with 20 wt % glycerol, the reactor was first heated to 80 °C before the nitrogen was displaced by oxygen. Once the desired temperature was reached, the pressure was increased to 5 bar by closing V-6 and further maintained by balancing inlet and outlet flows. The recording (oxygen concentration and filling level) for the fluid mechanical parameters started with a switch from nitrogen to oxygen aeration. The corresponding nozzle parameters and liquid volume flows together with the resulting specific energy dissipation rates are listed in Tables S4 and S5. Data recording was terminated once oxygen saturation in the liquid phase was detected by the oxygen probe (AIR O2) and afterward oxygen was stripped by aerating with nitrogen, allowing for new measurements once the oxygen concentration in the reactor dropped below 4 mg/L. Each measurement was conducted in duplicate, recording the liquid level before and after aeration and pressure loss at the nozzle. An exemplary graph for the oxygen concentration is shown in Figure S14. The data was used to determine the specific mass transfer coefficient *k*_l_ · *a*, gas hold-up ϵ_g_, and specific energy dissipation ε (Tables S6 and S7). The *k*_l_ · *a* value was calculated by eq 1, considering the oxygen saturation concentration *c*_g_^*^, the liquid volume flow *V̇*_l_, the reaction liquid volume *V*_l_, the oxygen concentration *c*_l,0_ at t = 0 s and the time dependent oxygen concentration *c*_l_(*t*) shown in Figure S15.^53–56^

$$k_{l}\cdot a=\ln\left(\frac{c_{g}^{*}-c_{l,0}}{c_{g}^{*}-c_{l}\left(t\right)}\right)\cdot \frac{{\mathrm{V̇}}_{l}}{V_{l}}$$ 1

For calculation of the specific energy dissipation rate ε, which represents the energy introduced into the JLR via the nozzle, the nozzle exit velocity ω_N. i_ is required. This was calculated using eq 2.

$$\omega_{N.i}=\frac{{\mathrm{V̇}}_{N,i}}{A_{N,i}}$$ 2

*V̇*_N, i_ is the volume flow of component i and *A*_N, i_ is the cross-sectional area of the nozzle opening in eq 2. The cross-sectional area *A*_N, l_ was determined according to eq 3.

$$A_{N,l}=\frac{\pi }{4}\cdot \left(d_{N}^{2}-d_{C}^{2}\right)$$ 3

The circular area is determined by subtracting the cross-section of the gas-carrying capillary from the total area of the nozzle and determining the outlet area as the annular gap. In eq 3, *d*_N_ is the diameter of the nozzle and *d*_C_ is the diameter of the gas-carrying capillary. The dimensions of the two-phase nozzle can be found in Table S4.^57^ With the knowledge of the nozzle exit velocity *ω*_N. i_ the specific energy dissipation rate ε was calculated according to eq 4.

$$\varepsilon =\frac{P}{V}=\frac{\sum {Ė}_{\mathrm{Kin}}}{V_{l}}=\frac{\rho_{l}\cdot {\mathrm{V̇}}_{l}\cdot \omega_{N,l}^{2}}{2\cdot V_{l}}+\frac{\rho_{g}\cdot {\mathrm{V̇}}_{g}\cdot \omega_{N,g}^{2}}{2\cdot V_{l}}\approx \frac{\rho_{l}\cdot {\mathrm{V̇}}_{l}\cdot \omega_{N,l}^{2}}{2\cdot V_{l}}$$ 4

Equation 4 shows that the sum of the kinetic energy ∑*Ė*_Kin_ introduced is equal to the kinetic energy introduced by the liquid phase. The density of the gas phase *ρ*_g_ is generally three to 4 orders of magnitude lower than the density of the liquid phases, therefore the term for the kinetic energy introduced by the gas phase can be considered negligible. The specific energy dissipation rate ε can be used to describe the existing flow regime and, by changing its value, influence the mixing of the phases, the interfacial area and the mass transfer.^57^ A further characterization parameter for JLRs is the gas hold up ϵ_g_. The gas hold-up ϵ_g_ was determined by using eq 5.^58^

$$\epsilon_{g}=\frac{V_{g}}{V_{l}+V_{g}}$$ 5

In eq 5, *V*_g_ is the gas volume and *V*_l_ is the liquid volume.^59^ The results for the gas hold-up obtained can be found in the diagrams in Figure S13 and Figure 3.

**Figure 3:**
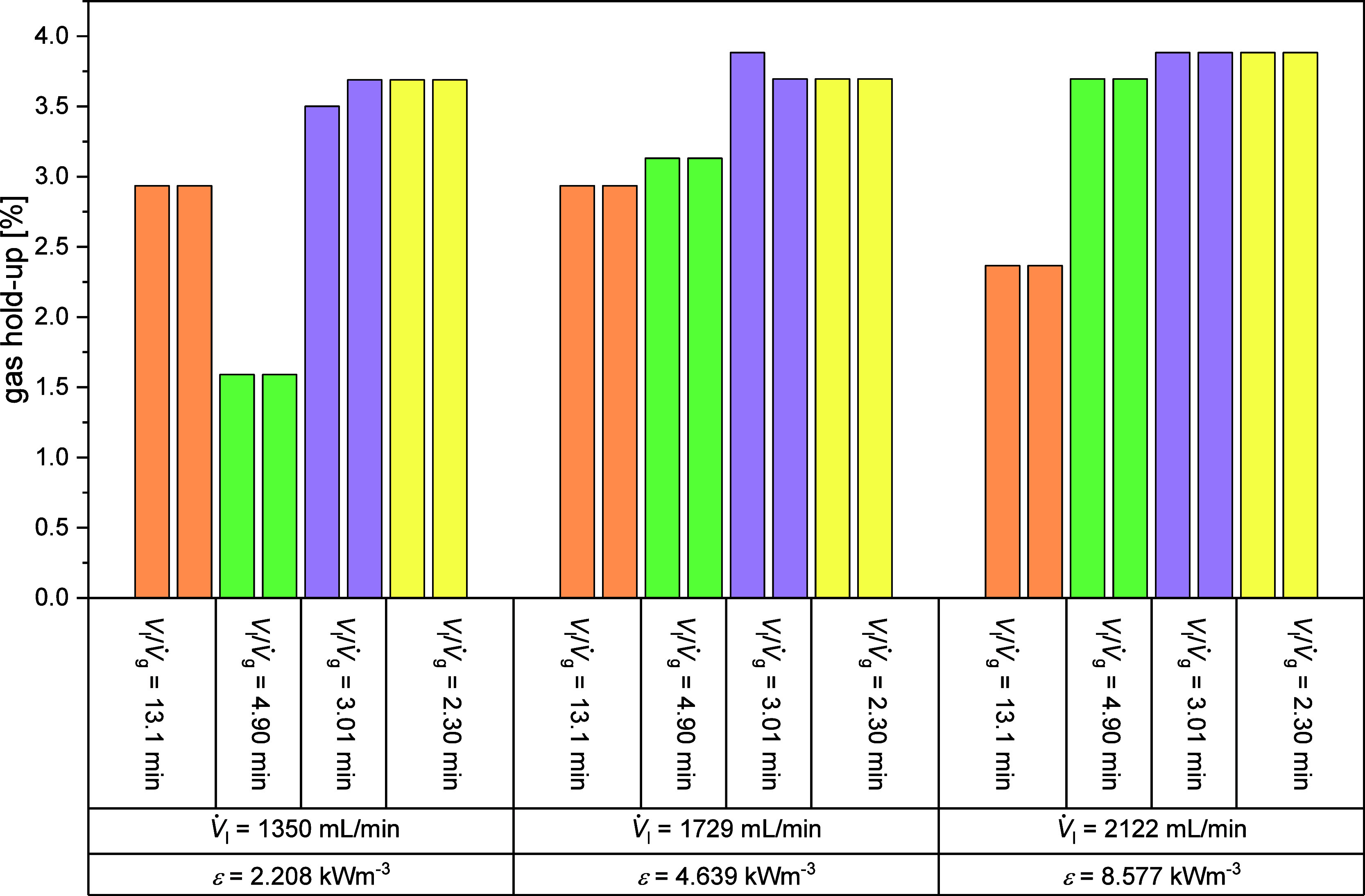
Gas hold-up over specific energy dissipation rate for different gas- and liquid flows (*p* = 5 bar_Oxygen_, *T* = 80 °C, 20 wt % Glycerol in water).

### Catalytic Experiments in the JLR

The reference experiments for the selective oxidation of glycerol in the JLR were conducted with a liquid flow rate of 1729 mL/min, a gas flow rate of 650 N mL/min, a temperature of 115 °C, a total system pressure of 5 bar oxygen, a reaction time of 6 h, and a catalyst concentration of 5 mmol/L HPA-2. To provide statistical significance using different batches of HPA-2, a reference experiment with each new batch of HPA-2 was carried out, each time using the same amount of vanadium (calculated from ICP-OES, Table S2) to ensure overall comparability. The reactor was filled with 10 wt % aqueous glycerol to approximately 1960 mL (point 1 on the sight glass), with the exact level noted and volume determined via a linear plot (Figure S16). Two stock solution samples were taken. The substrate solution was circulated and heated to 90 °C, with oxygen flow set to 150 N mL/min, and the outlet flow matched the inlet at atmospheric pressure. The catalyst was dissolved in 50 mL demineralized water. When the temperature reached 90 °C and oxygen atmosphere was established, the aqueous catalyst solution was added to the JLR by opening V-5. Afterward, an oxygen pressure of 3 bar was applied with 650 N mL/min V̇_g_, and the temperature was set to the desired reaction temperature of 115 °C. Oxygen concentration, exhaust flow, and gas analyses (CO, CO_2_ and O_2_) were recorded. Samples were taken initially every 5 min for 30 min, then every 15 min, and every 30 min after 2 h and were cooled down with ice to quench the reaction. The start of the reaction was marked when the temperature reached 115 °C, and oxygen pressure was increased to 5 bar and maintained throughout. Postreaction, liquid samples were filtered through a syringe and analyzed by High Performance Liquid Chromatography (HPLC). To determine the kinetic parameters of the selective glycerol oxidation, additional experiments with varied reaction conditions were carried out. This included experiments with 5 and 20 wt % glycerol to determine the reaction order of glycerol, experiments at different oxygen partial pressures *p*_Oxygen_ = 1.04 bar and *p*_Oxygen_ = 1.96 bar to determine the reaction order of oxygen and experiments with temperatures of 100 °C, 104 °C, 108 °C, and 112 °C to determine the activation energy. To analyze whether the reaction is limited by mass transfer, a mass transfer limitation experiment was performed at maximum liquid (2122 mL/min) and gas (850 N mL/min) gas flow rates and compared to the reference experiments. To check the stability of the catalyst, an experiment was conducted over 3 days, operating for 6 h each day with 5 wt % glycerol. Each day, the amount of glycerol converted was added back to the JLR.

### Catalytic Experiments in the STR

To evaluate the performance of the JLR for the glycerol oxidation to FA, comparative tests were conducted in a classical STR having the same reaction volume shown in Figure S17. The experiments in the STR were conducted in the same manner as the reference experiments in the JLR, using the following reaction conditions: 1729 N mL/min liquid flow rate, stirring rate of 1200 or 1800 rpm, a temperature of 115 °C, a process pressure of 5 bar oxygen, a reaction time of 6 h after catalyst addition, and a catalyst concentration of 5 mmol/L HPA-2. For this purpose, a high-pressure oxidation plant with a gas-entrainment stirrer and a stirrer tank reactor (STR) (2.00 L nominal volume, 1.90 L working volume and 1.60 L liquid volume) was used. The reactor vessel and the gas-entrainment stirrer are made of Hastelloy C276, while all pipes, valves, and fittings were made of stainless steel 1.4571. The flowsheet of the plant can be found in Figure S17. The reactor was filled with 1.6 L of 10 wt % aqueous glycerol solution and the experiments were conducted analogously to the reference experiments in the JLR. The gas flows were also controlled using MFCs by Bronkhorst. The temperature was regulated via cascade control using the Flexlab software. Gas analysis was conducted through half-hourly gas samples taken in vacuumed gas bags via valve V-17. The gas samples were measured using a Varian 450-GC offline gas chromatograph equipped with a Shin-Carbon-ST-Column (2 m x 0.75 mm). Quantification for CO, CO_2_ and O_2_ content was carried out using stored calibration data of the pure substances and performed using Galaxy chromatography Data systems software.

### Calculation of Kinetic Parameters in the JLR

For the experiments carried out in the JLR, all liquid products were quantitatively analyzed using HPLC and all gaseous products were quantitatively analyzed by online Infrared spectroscopy (IR). HPLC liquid phase analysis was carried out in a Nexera 40 from Shimadzu equipped with a polymer phase organic acid column (300 mm × 8 mm) from Chromatographie-Service GmbH. The eluent was aqueous sulfuric acid (4 mmol/L) at a flow rate of 0.8 mL/min at 25 °C. The detection sensor after the column was a Refractive Index Detector (RID). All possible products and intermediates have been previously calibrated. A sample chromatogram and the respective retention times of all intermediates and products is shown in Figure S18 and Table S8. Curves for HPLC calibration are shown in Figures S19–S23.

The conversion *X*_Glycerol_ was determined from the HPLC results of the liquid phase according to eq 6.

$$X_{\mathrm{Glycerol}}\left[\%\right]=\left(\frac{n_{\mathrm{Glycerol},0}-n_{\mathrm{Glycerol}}}{n_{\mathrm{Glycerol},0}}\right)\cdot 100=\left(\frac{c_{\mathrm{Glycerol},0}-c_{\mathrm{Glycerol}}}{c_{\mathrm{Glycerol},0}}\right)\cdot 100$$ 6

In eq 6, *n*_Glycerol,0_ is the amount and *c*_Glycerol,0_ the concentration of glycerol at time zero before the start of the reaction and *n*_Glycerol_ is the amount and *c*_Glycerol_ the concentration of glycerol at the respective time during or after the reaction. The ideal gas equation according to eq 7 was used to convert the measured volume percentages *V* – % by the online IR device into mass fraction *n*_i_.

$$n_{i}=\left(\frac{p_{\mathrm{removal}}\cdot V_{R}}{R\cdot T_{\mathrm{removal}}}\right)\cdot V-\%$$ 7

$$n_{i}=\left(\frac{p_{G}\cdot {\mathrm{V̇}}_{G}}{R\cdot T_{G}}\right)\cdot V-\%$$ 8

In eq 7, R is the general gas constant (8.314 J · (mol ·K)^−1^), *V̇*_G_ is the volume flow, *p*_G_ is the pressure, *V*_R_ is the gas volume of the reactor and *T*_G_ is the temperature of the gas phase flowing through the online IR device. With the determined amount of substance of the gas phase and the measured amount of substance of the liquid phases by HPLC, the respective yield *Y*_i_ can be determined according to eq 9.

$$Y_{i}\left[\%\right]=\left(\frac{n_{i}\cdot \sum C-{\mathrm{Atoms}}_{i}}{n_{\mathrm{Glycerol},0}\cdot \sum C-{\mathrm{Atoms}}_{\mathrm{Glycerol}}}\right)\cdot 100=\left(\frac{c_{i}\cdot \sum C-{\mathrm{Atoms}}_{i}}{c_{\mathrm{Glycerol},0}\cdot \sum C-{\mathrm{Atoms}}_{\mathrm{Glycerol}}}\right)\cdot 100$$ 9

In eq 9, ∑C – Atoms_i_ is the sum of the C atoms of product i, ∑C – Atoms_Glycerol_ is the sum of the C atoms of glycerol and *n*_i_ is the amount and *c*_i_ the concentration of product i.

The selectivity *S*_i_ of each product i is determined from the ratio of the yield *Y*_i_ of product i to the conversion of glycerol *X*_Glycerol_. This is given by eq 10.

$$S_{i}\left[\%\right]=\left(\frac{Y_{i}}{X_{\mathrm{Glycerol}}}\right)\cdot 100$$ 10

In order to ensure comparability with other reactors, a key parameter was introduced: the space-time yield (STY). STY is the performance of a reactor (mass of FA *m*_FA_ product) in relation to the volume of the reaction volume *V*_R_ used in a specific time period *t*. The STY is determined according to eq 11.

$$\mathrm{STY}=\frac{m_{\mathrm{FA}}}{V_{R}\cdot t}$$ 11

The arithmetic mean x̅ and the standard deviation σ are calculated for several tests with exactly the same conditions according to eqs 12 and 13.

$$\overset{¯}{x}=\frac{1}{n}\overset{n}{\underset{i=1}{\sum }}x_{i}$$ 12

$$\sigma =\sqrt{\frac{\overset{n}{\underset{i=1}{\sum }}{\left(x_{i}-\overset{¯}{x}\right)}^{2}}{n-1}}$$ 13

In eqs 12 and 13, *n* is the number of trials and *x*_i_ is the measured value in the respective trial *i*.

To determine the kinetic parameters of the reaction, the general rate law of all chemical reactions is given in eq 14 using the exponential approach.

$$r\left(T,c\right)=\frac{dc_{i}}{dt}=k\left(T\right)\cdot \overset{i}{\underset{i=1}{\prod }}c_{i}^{n_{i}}$$ 14

In eq 14, *r* (T, c) is the temperature and concentration dependent reaction rate, c_i_ is the respective concentration of a reactant i, *k*(T) is the temperature dependent rate constant and *n*_i_ is the reaction order of the respective partial reaction. In the selective glycerol oxidation carried out in this work, two partial reactions take place in parallel. One is the oxidation of the starting material glycerol, and the other is the reoxidation of the catalyst by oxygen. The catalytic cycle is shown in the graphical abstract. The rate laws are given by eqs 15 and 16.

$$r_{1}=k_{1}\cdot c_{Glycerol}^{a}\cdot c_{HPA-2_{\mathrm{ox}}}^{b}$$ 15

$$r_{2}=k_{2}\cdot c_{O_{2}}^{c}\cdot c_{HPA-2_{red}}^{d}$$ 16

The simplified representation of the rate laws where the latter are only dependent on the concentration of the reactants can be seen in eqs 17 and 18.

$$r_{1}=k_{1}^{\prime }\cdot c_{Glycerol}^{a}$$ 17

$$r_{2}=k_{2}^{\prime }\cdot c_{O_{2}}^{c}$$ 18

$$because\;\frac{dc_{HPA-2}}{dt}\approx 0$$ 19

$$with\;k^{\prime }=k\cdot c_{HPA-2}^{b,d}$$ 20

By applying the natural logarithm to eqs 17 and 18, eq 21 is obtained. This allows for a linearization approach.

$$\ln\left(r\right)=\ln\left(k^{\prime }\right)+a\cdot \ln\left(c_{0,i}\right)$$ 21

The order of the reaction can be deduced from the slope a. The rate constant is determined from eq 22.

$$k^{\prime }=\frac{r}{c_{0}^{x}}$$ 22

In eq 22, *c*_0_^x^ is the concentration at time zero.

The activation energy can be determined using the rate constants and the Arrhenius plot, as well as a linear regression through these (see eqs 23 and 24).

$$k\left(T\right)=A\cdot e^{\left(-E_{A}/R\cdot T\right)}$$ 23

$$\ln\left(\frac{k\left(T\right)}{A}\right)=-\frac{E_{A}}{R}\cdot \frac{1}{T}$$ 24

In eq 23, A is the pre-exponential factor. Equation 20 can be converted to eq 25. If the reaction order of the catalyst is known, the reaction constant *k* can be determined using the converted equation.

$$k=\frac{k^{\prime }}{c_{\mathrm{HPA}-2}^{b,d}}$$ 25

The reaction constant *k* can be used to determine the Hatta number (*Ha*) of the chemical reaction according to eq 26. The Hatta number can be used to categorize the reaction and compare it with others.

$$Ha=\delta_{l}\sqrt{\frac{k\left(c_{O_{2}}^{n-1}\right)}{D_{O_{2},l}}}$$ 26

$$\mathrm{with}\;\delta_{l}=\frac{D_{O_{2},l}}{k_{O_{2},l}}$$ 27

$$\mathrm{with}\;k_{O_{2},l}=\frac{k_{l}a}{a}$$ 28

$$\mathrm{with}\;a=\frac{6\varepsilon_{g}}{d_{\mathrm{mean}}}$$ 29

In eqs 26 to 29, δ_l_ is the thickness of the liquid side boundary layer, *D*_*O*_2_, *l*_ is the diffusion coefficient of oxygen in the liquid phase, *k*_O_2_, l_ is the mass transfer coefficient of oxygen, a is the total bubble surface area and *d*_mean_ is the Sauter mean diameter.

## Results and Discussion

### Investigation of the Hydrodynamics and Oxygen Solubility in the JLR: Specific Energy Dissipation Rate, Volumetric Mass Transfer Coefficient, and Gas Hold-up

The macro mixing and fluid dynamics of a specific reactor design are of special interest for the application for a precise reaction. One advantage is the generation of a large interfacial surface area, which favors mass transfer from the gas to the liquid phase in multiphasic systems. The mass transfer is supported by higher gas hold-ups and characterized by the volumetric mass transfer coefficient *k*_l_ *a*.^60–62^ In the following, the JLR is characterized for its hydrodynamic parameters using a 20 wt % glycerol in water mixture. Knowledge obtained about the mass transfer of oxygen into the liquid phase in dependence of the applied reaction conditions in the JLR can be applied to the catalytic oxidation process.

In Figure 3 the gas hold-up calculated by eq 5 for each experiment is plotted against the specific energy dissipation rate ε. For the values 4.90 min, 3.01 min, 2.30 min of *V*_l_ */V̇*_g_, the gas flow rate appears to have a greater influence on the gas content. Gas volumetric flow rates that are on the higher end (850 N mL/min) lead to higher achieved gas hold-ups (3.9%). The volumetric mass transfer coefficient *k*_l_ · *a* as a measure for process intensification was determined by the slope of the linear regression over the linearized values of the measured oxygen concentrations (Figures S14–S15). In this way, the *k*_l_ · *a* values for all parameter combinations in the reactor characterization experiments could be obtained. For the determination of the *k*_l_ · *a* values, experiments were conducted at room temperature with water and at 80 °C with 20 wt % glycerol. In Figure 4 all calculated volumetric mass transfer coefficients *k*_l_ · *a* are plotted against the specific energy dissipation rate ε at room temperature with water. The *k*_l_ · *a* values determined at 80 °C with 20 wt % glycerol can be found in Figure S16. The latter was calculated by using eq 4 and the included nozzle exit velocity was deduced from eq 2 for the corresponding liquid volume flow. The specific energy dissipation rate induced by the nozzle in the liquid phase and the associated outlet velocity of the liquid phase, as well as the dimensions of the nozzle are shown in Tables S4–S7.

**Figure 4:**
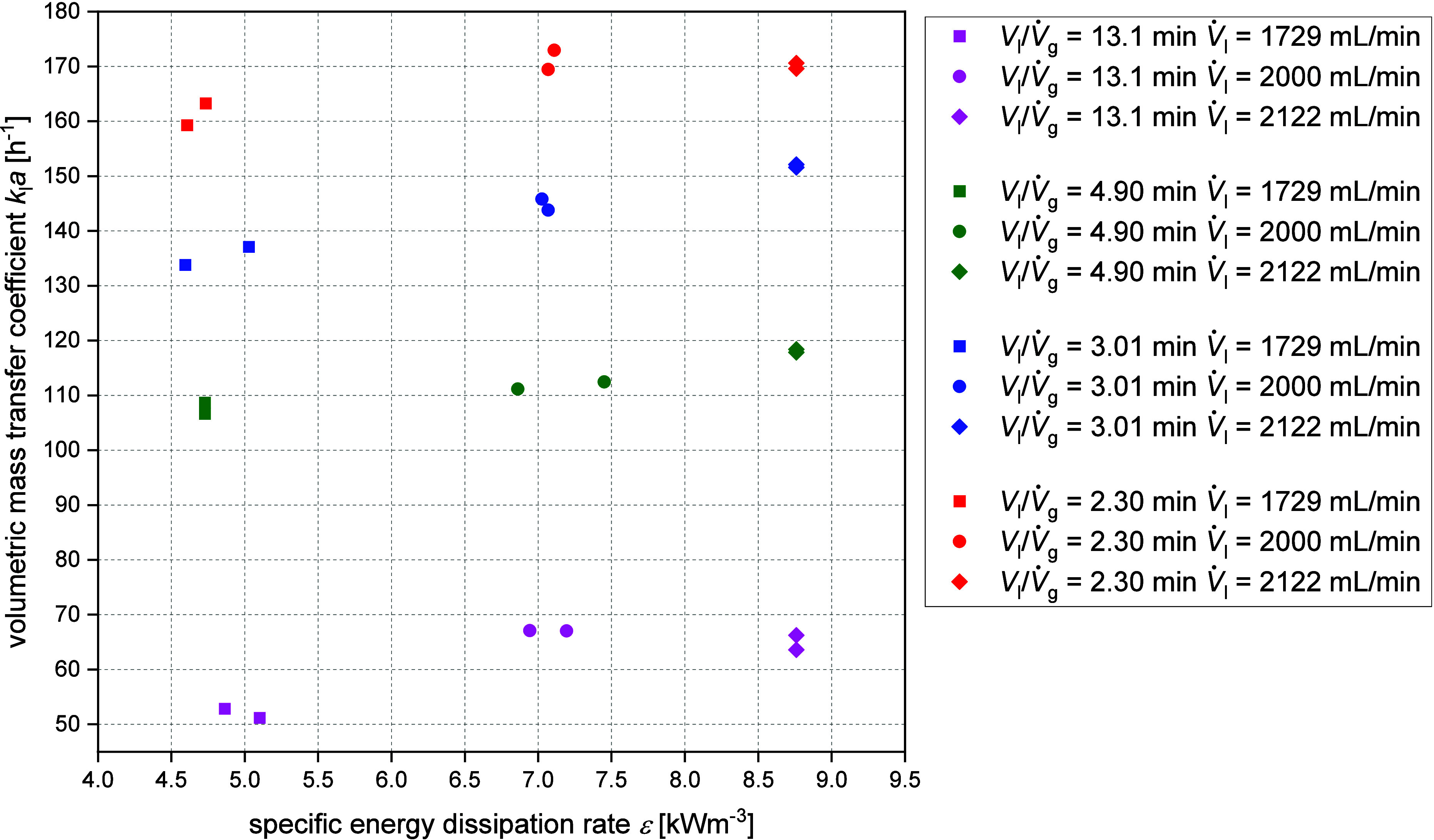
Volumetric mass transfer coefficient plotted over specific energy dissipation rate for different gas- and liquid flows (square: liquid flow *V̇*_l_ = 1729 $\frac{\mathrm{mL}}{\min}$ , circle: liquid flow *V̇*_l_ = 2000 $\frac{\mathrm{mL}}{\min}$ , rhombus: liquid flow *V̇*_l_ = 2122 $\frac{\mathrm{mL}}{\min}$ , *p* = 5 bar_Oxygen_, *T* = 80 °C, 20 wt % Glycerol in water).

Interestingly, the volumetric mass transfer coefficient *k*_l_ · *a* does not change significantly as the specific energy dissipation rate ε increases, but it appears to run toward saturation. There is a tendency for *k*_l_ · *a* to decrease from 173 h^–1^ to 51.2 h^–1^ at larger values for the ratio of reactor liquid volume to gas volume flow (from 2.3 to 13.1 min). The ratio decreases at lower gas volume flows, so the *k*_l_ · *a* value is more dependent on the gas flow rate *V̇* *_g_*. The maximum determined value of 173 h^–1^ for the volumetric mass transfer coefficient *k*_l_ · *a* is at the maximum set values for the oxygen and liquid flow (*V*_l_/*V̇*_g_: 2.30 min, *V̇*_g_ = 850 N mL/min and *V̇*_l_ = 2122 mL/min). The presented data exhibit the same trend as the results obtained by Maly et al.,^57^ who hydrodynamically characterized a JLR reactor identical in construction using an air–water system. The *k*_l_ · *a* value becomes smaller at larger numbers for the ratio of reactor liquid volume to gas volume flow (*V*_l_/*V̇*_g_). The ratio decreases at lower gas volume flows, so the *k*_l_ · *a* value is more dependent on the gas flow rate. Compared to the maximum *k*_l_ · *a* value of around 90 h^–1^ of Maly et al.^57^ for a pure aqueous system,^57^ the obtained *k*_l_ · *a* values in this work are significantly higher. This difference speaks in favor of the system used in this work, which achieved higher fluid flow rates (*V̇*_l_) and, consequently, higher specific energy dissipation rates ε (see eq 2 and ([Disp-formula eq4])). Another possible reason is the higher oxygen pressure of 5 bar. The oxygen saturation is pressure-dependent and becomes higher at advanced pressures.^63^

### Selective Catalytic Oxidation of Glycerol in the JLR

After the JLR reactor has been characterized for its hydrodynamic parameters and mass transfer properties, the next objective was to find out how the JLR performs in comparison to the classical STR under similar reaction conditions. For the implementation of the selective oxidation of glycerol to FA, the experiments were carried out as described in the experimental section. The JLR can achieve volumetric mass transfer coefficients *k*_l_ · *a* ranging from 51 h^–1^ and 173 h ^–1^. For the reference experiments in Figure S24, the *k*_l_ · *a* value was set to 152 h^–1^ and the ε value to 4.67 kW ·m^–3^. These values are in the upper half of the middle range of the possible achievable values within the JLR. With values selected in the middle range, it was possible to conduct an additional experiment with maximum *k*_l_ · *a* value and ε value. Increasing the *k*_l_ · *a* value increases the mass transfer coefficient *k*_l_ and the specific interface *a*, which could accelerate the conversion in a mass-transfer-limited reaction. The additional experiment was conducted to check the system for potential mass transfer limitations. However, the results showed no differences in conversion (Figure S25) clearly showing the absence of such limitations.

The simplified reaction scheme is shown in Figure 5. Herein, glycerol is first oxidized to glyceric aldehyde and further to glyceric acid. Alternatively, glyceric aldehyde can undergo isomerization to dihydroxyacetone that is further oxidized to hydroxyacetone or undergo direct C–C bond cleavage to glycol aldehyde. Afterward, the C_3_-compounds split into C_2_-compounds (glycolic acid, acetic acid or glyoxal) and further to the respective C_1_-compounds FA as the desired product or CO_2_ and CO.

**Figure 5:**
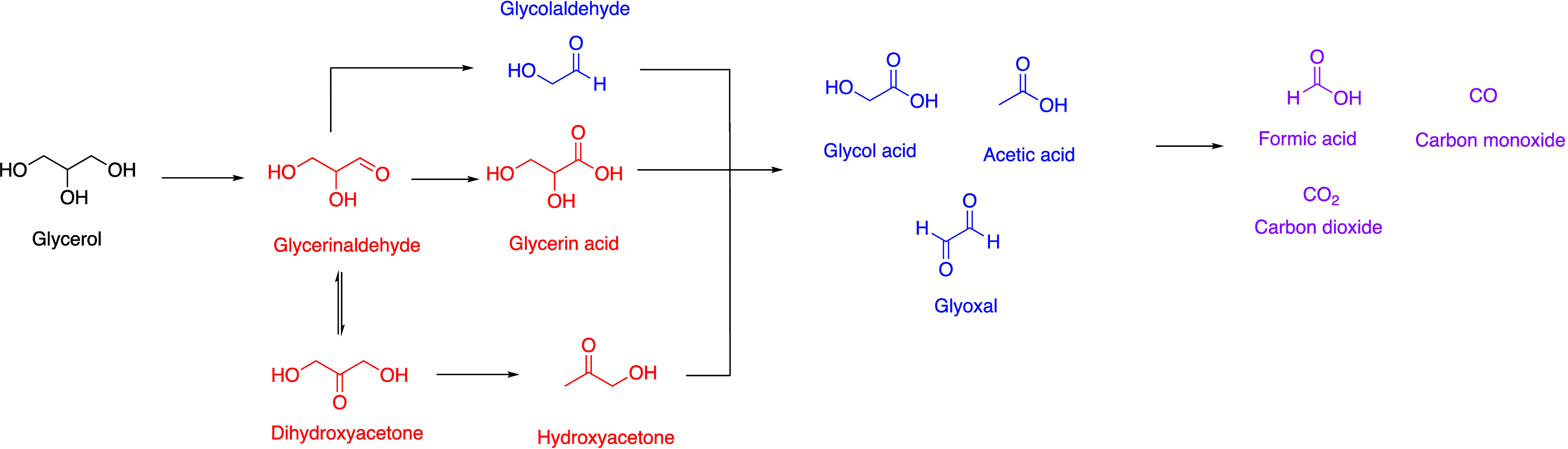
Simplified reaction network of selective glycerol oxidation to formic acid (Red: C_3_ Intermediates, Blue: C_2_ Intermediates and Pink: C_1_ Products) based on the illustration of Katryniok et al.^7^

The averaged STY of the three reference experiments (Figure S24) show a good reproducibility in the JLR (Table S9). That speak in favor of the robustness and successful implementation of the selective glycerol oxidation to FA in the JLR. In order to compare the performance of the JLR with the STR, the reaction was also carried out in the STR under the same reaction conditions as described in the corresponding part of the experimental section. Figure 6 shows the comparison of the two different reactor concepts under identical reaction conditions. Herein, a maximum STY of 26.9 g_Fa_ L_R_^–1^ h^–1^ was achieved in the JLR at short reaction times decreasing down to a value of 14.0 g_Fa_ L_R_^–1^ h^–1^ at the end of the reaction. At the beginning of the reaction in the STR at 1200 rpm, the STY is at 18 g_Fa_ L_R_^–1^ h^–1^ compared to the STY of the JLR being at 26.9 g_Fa_ L_R_^–1^ h^–1^ with a standard deviation of 1.24 g_Fa_ L_R_^–1^ h^–1^. As reaction time increases, standard deviation decreases because FA is an end product of glycerol oxidation, and the reaction is nearly complete after two and a half hours. The STY values achieved in the JLR exceed the STY of the STR by a factor of 1.5 at the beginning of the reaction. The large initial difference, which equalizes after around 3 h, clearly demonstrates the JLR’s excellent mixing properties and gas input. These properties favor oxygen mass transfer into the liquid phase. The convergence of the curves after 3 h is due to the depletion of the glycerol reactant in the reaction system, as evidenced by the conversion curve reaching a plateau (see Figures S25 and S26 for the averaged reference experiments in the JLR and STR). In summary, the JLR outperforms the STR in terms of STY by a factor of 1.5, supporting a successful process intensification from a classically used STR in the more economical reactor concept of the JLR.

**Figure 6:**
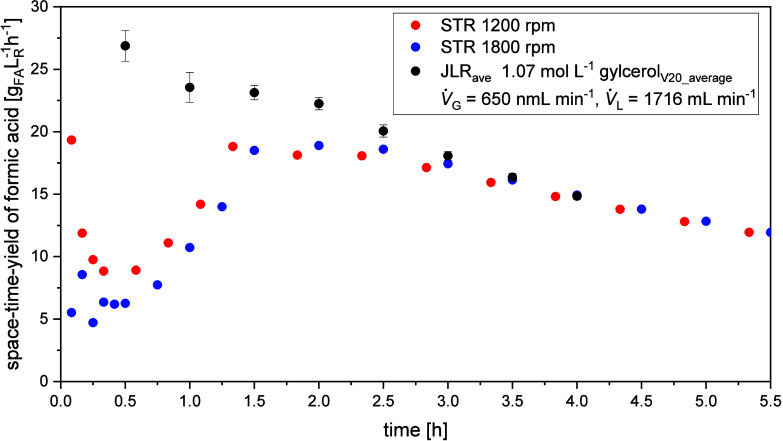
Average of the STY for FA (black) versus reaction time for the three reference experiments in the JLR including standard deviation (*V̇*_g_ = 650 $\frac{\mathrm{NmL}}{\min}$ , *V̇*_l_ = 1729 $\frac{\mathrm{mL}}{\min}$ , *p*_O_2__ = 5 bar, *t* = 115 °C, *c*_0_ = 10 wt % Glycerol, *c*_Cat_ = 5 $\frac{\mathrm{mmol}}{L}$ HPA-2, *t* = 6 h). In red and blue the STY for the two experiments in the stirred tank reactor (V̇_g_ = 650 $\frac{\mathrm{NmL}}{\min}$ , stirrer speed = 1200 or 1800 rpm, *p*_O_2__ = 5 bar, *t* = 115 °C, *c*_0_ = 10 wt % Glycerol, *c*_Cat_ = 5 $\frac{\mathrm{mmol}}{L}$ HPA-2, *t* = 6 h).

Having a closer look into the cumulative yield and selectivity of the intermediates and reaction products during the course of the reaction, no significant differences between the two reactor concepts could be revealed (Tables S10–S13). Figure 7 shows the cumulative yield and selectivity of all intermediates and products in the JLR with a constant increase in FA yield up to 40% after 6 h, whereby the selectivity reaches a plateau of 51–53% after already 3 h reaction time. The intermediates glyceraldehyde, hydroxyacetone, glycolaldehyde, glycolic acid, glyoxal and acetic acid are first formed after 1–3 h reaction time and afterward consecutively converted to either FA or CO_2_. The amount of CO is always very low at around 1.5%.

**Figure 7:**
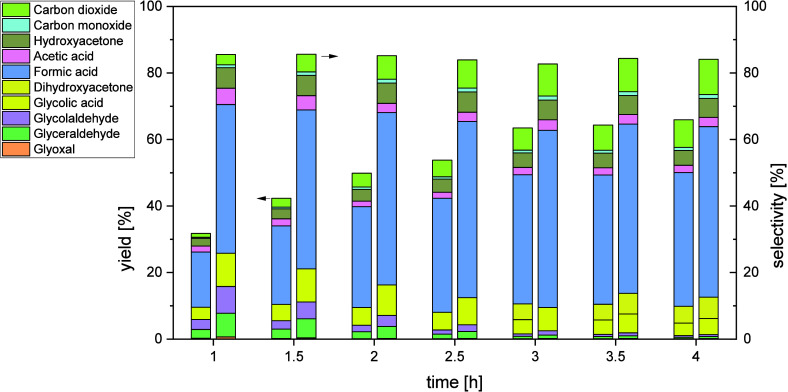
Cumulative yields (left) and selectivities (right) of intermediates and products in the JLR with time-on-stream. Reaction conditions: *V̇*_g_ = 650 $\frac{\mathrm{NmL}}{\min}$ , *V̇*_l_ = 1729 $\frac{\mathrm{mL}}{\min}$ , *p* = 5 bar_Oxygen_, *T* = 115 °C, *c*_Substrate_ = 10 *wt.*% glycerol, c_catalyst_ = 5 $\frac{\mathrm{mmol}}{L}$ HPA-2, *t* = 6 h.

In the STR (Figure 8), the yield of FA also increases with prolonged reaction time up to 41% after 6 h with a constant selectivity of around 48–50%. The behavior after 3 h is similar to the JLR with no further increase in FA yield. Moreover, the formation and depletion of the intermediates follows the same trend as in the JLR. In summary, there is no significant difference between the two reactor types with respect to FA yield and selectivity, however higher STY could be achieved in the JLR due to the improved mass transfer.

**Figure 8:**
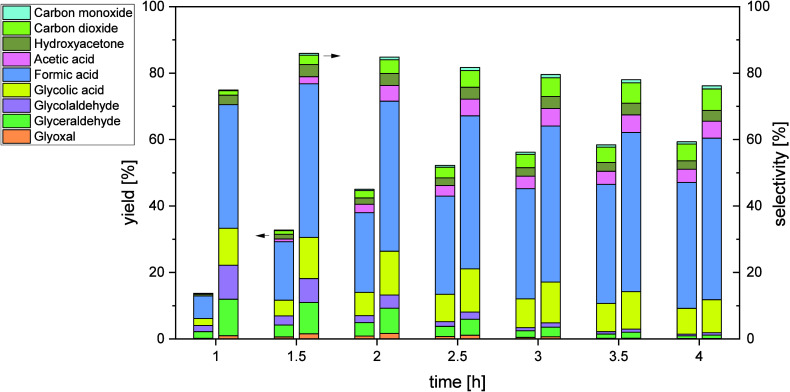
Cumulative yields (left) and selectivities (right) of intermediates and products in the STR with time-on-stream. Reaction conditions: *V̇*_g_ = 650 $\frac{\mathrm{NmL}}{\min}$ , *n* = 1800 rpm, *p* = 5 bar_Oxygen_, *T* = 115 °C, c_Substrate_ = 10 wt % glycerol, *c*_catalyst_ = 5 $\frac{\mathrm{mmol}}{L}$ HPA-2, *t* = 6 h.

Before the kinetic parameters for the JLR could be determined, we had to clarify that the reaction is in its kinetic regime and no longer influenced by mass transfer effects. Therefore, an additional experiment was conducted applying the maximum achievable values for *k*_l_ · *a* and ε in the JLR by setting maximum flow rates for gas and liquid in order to maximize the mass transfer between the gas phase and the liquid phase and in turn the entrainment of oxygen into the liquid phase. To compare the results for glycerol conversion of the experiment with the maximum *k*_l_ · *a* and ε values to the reference experiment, the glycerol conversion of the three reference experiments was averaged and the standard deviation was determined according to eqs 12 and ([Disp-formula eq13]) and plotted against the reaction time. The conversion of the experiment with the maximum values was calculated according to eq 6. The results were plotted against reaction time in Figure 9.

**Figure 9:**
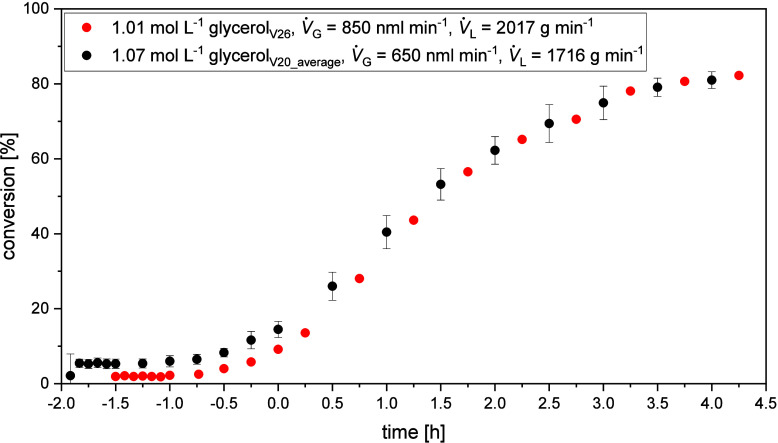
Plot of the averaged conversion of the three reference experiments in the JLR including standard deviation against reaction time (*V̇*_g_ = 650 $\frac{\mathrm{NmL}}{\min}$ , *V̇*_l_ = 1729 $\frac{\mathrm{mL}}{\min}$ , *p* = 5 bar_Oxygen_, *T* = 115 °C, *c*_Substrate_ = 10 wt % glycerol, *c*_catalyst_ = 5 mmol/L HPA-2, *t* = 6 h), and plot of the conversion of maximum energy input experiment against reaction time in the JLR. (*V̇*_g_ = 850 $\frac{\mathrm{NmL}}{\min}$ , *V̇*_l_ = 2122 $\frac{\mathrm{mL}}{\min}$ , *p* = 5 bar_Oxygen_, *T* = 115 °C, *c*_Substrate_ = 10 wt % glycerol, *c*_catalyst_ = 5 mmol/L HPA-2, *t* = 6 h).

The conversion of the experiment with maximum *k*_l_ · *a* value and ε value in the JLR are within the standard deviation of the reference experiments. Therefore, the reaction cannot by further accelerated by increasing the mass transfer concluding that it already operates in its kinetic regime where the gas entrainment and therefore catalyst reoxidation is faster than the substrate oxidation. In relation to the classical OxFA process operating in a STR, where, according to Reichert et al.,^36^ no mass transfer limitation is only present at pressures above 30 bar, the reaction in the JLR achieves higher STY at a significantly lower oxygen pressure of only 5 bar, supporting the concept of the JLR with improved mass transfer capabilities.

### Determination of Kinetic Parameters for Selective Glycerol Oxidation in the JLR

In order to compare the kinetics of the glycerol oxidation in the JLR in relation to other multiphasic oxidation reactions, the Hatta number was determined using eqs 26 to ([Disp-formula eq28]). To determine the *k* value according to eq 25, a value of 0.98 was used for the reaction order of HPA-2, which was determined by Ponce et al.^41^ The value for the mean Sauter diameter (*d*_mean_) required for the calculation was determined in an identical reactor for a water–air system by Maly et al.^57^ to be 0.013 m. The reaction order of oxygen was assumed as a pseudo first order at 5 bar total pressure according to Reichert et al.^36^ The values for calculating the Hatta number are given in Table S14. The Hatta number was calculated to 0.014, being clearly below 0.3, showing that the reaction is very slow. This combined with the efficient and fast mass transfer indicates that the reaction is only limited by kinetics in the JLR.

In the next step, we wanted to have a closer look on the relevant kinetic parameters for achieving explicit control of the reaction for possible industrialization and scale-up.^58^ One of the most important kinetic parameters is the reaction order of the reactants. With the purpose of determining the reaction orders of both reactants (glycerol and oxygen) independently, the initial reaction rates must first be determined according to eqs 15 and ([Disp-formula eq16]). Equations 15 and ([Disp-formula eq16]) were chosen based on studies conducted by Reichert et al.^36^ and Ponce et al.,^44^ where detailed kinetic investigations on various substrates were carried out, using the same HPA-2 catalyst.

### Reaction Order of Glycerol

It is noted that for the case of r_2_≫r_1_ the concentration of HPA-2 is in fact constant over time (see eq 19). This assumption can be done because no change in oxygen concentration is observed during the course of the reaction (see Figure S39). To determine the observed reaction rate (*r*_obs_), eq 17 was used in Figures S27–33. The glycerol concentration was normalized by dividing the time-dependent glycerol concentration c­(t) by the initial glycerol concentration (*c*_0_) and plotted against reaction time (*t*), with a linear regression through the points in the kinetic regime. Reaction rates (*r*_obs_) were determined in the same way for all six different initial glycerol concentrations (0.5–2.0 mol/L). To obtain the effective reaction rates (*r*_eff_), *r*_obs_ was multiplied by the initial glycerol concentration (*c*_0_). By linearizing these obtained values, logarithmizing and applying a linear regression over the points, the reaction order of glycerol (*n*_gly_) can be determined from the slope of the regression. This is shown in Figure 10.

**Figure 10:**
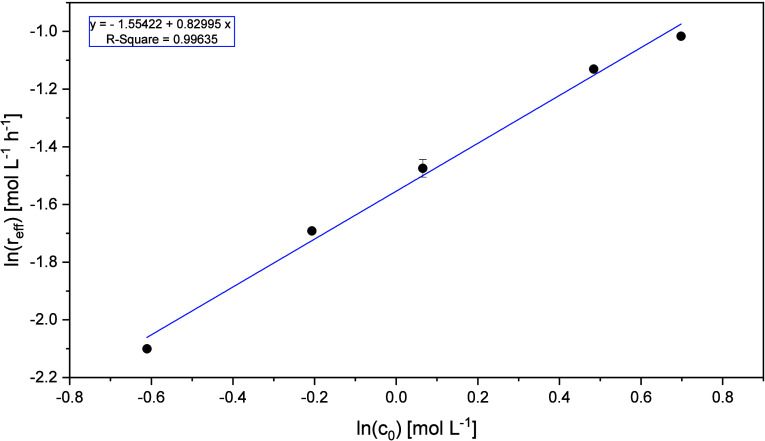
Plot of the natural logarithm of the effective reaction rates for various glycerol concentrations (incl. standard deviation) against the natural logarithm of the initial glycerol concentrations (*V̇*_g_ = 650 $\frac{\mathrm{NmL}}{\min}$ , *V̇*_l_ = 1729 $\frac{\mathrm{mL}}{\min}$ , *p* = 5 bar_Oxygen_, *t* = 115 °C, *c*_Catalyst_ = 5 $\frac{\mathrm{mmol}}{L}$ HPA-2, *t* = 6 h).

The slope of the linear regression in Figure 10 shows that the reaction order of glycerol is 0.83. Glycerol does not have an integer reaction order due to the fact that several subsequent oxidation steps take place. Combining the investigated glycerol oxidation steps the reaction order is made up of its partial reaction orders. With the reaction order of 0.83 for glycerol, eq 30 provides the rate law for glycerol as follows:

$$r_{1}=k_{1}^{\prime }\cdot c_{\mathrm{Glycerol}}^{0.83}$$ 30

The reaction order of 0.83 determined for glycerol in the JLR corresponds to previously determined reaction orders in the STR for various substrates used in the OxFA process.^36^

### Reaction Order of Oxygen

Analogous to the previous section, eq 31 was used to determine the reaction order of oxygen (Figure 11) during catalyst reoxidation (V^4+^ → V^5+^). In addition to the three reference experiments with 100 V.-% oxygen (*p*_*O*_2__ = 5 bar), two further experiments were carried out with 20.88 *V*-% (air, *p*_*O*_2__ = 1.04 bar) and 39.25 V-% (*p*_*O*_2__ = 1.96 bar) oxygen (Figures S36–S38). All other parameters were kept constant throughout the experiments. The normalized glycerol concentration versus the reaction time with the linear regression can be found in Figures S34–35.

**Figure 11:**
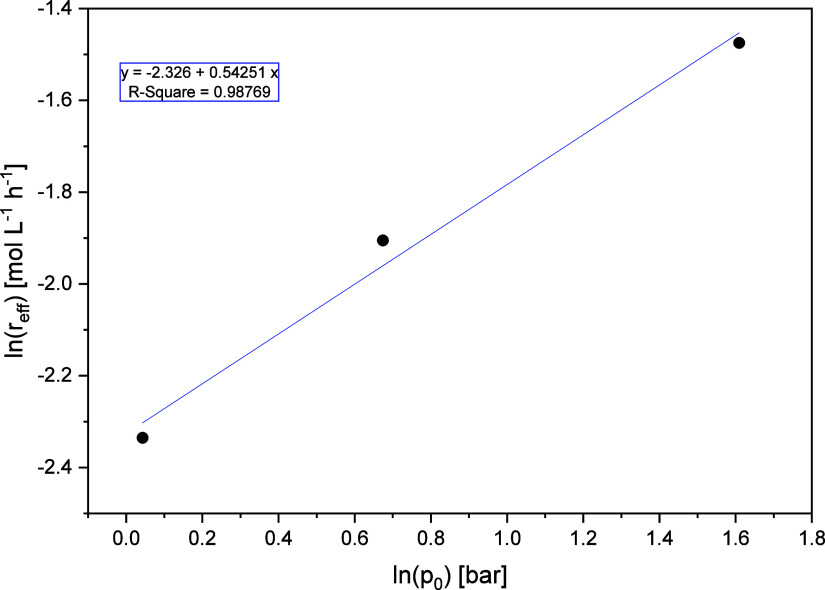
Plot of the natural logarithm of the effective reaction rates for various initial oxygen pressures (*V̇*_g_ = 650 $\frac{\mathrm{NmL}}{\min}$ , *V̇*_l_ = 1729 $\frac{\mathrm{mL}}{\min}$ , *c*_Substrate_ = 10 wt % glycerol, *t* = 115 °C, *c*_Catalyst_ = 5 $\frac{\mathrm{mmol}}{L}$ HPA-2, *t* = 6 h).

The slope of the linear regression in Figure 11 results in a reaction order of 0.54, therefore the rate law for oxygen is

$$r_{2}=k_{2}^{\prime }\cdot c_{O_{2}}^{0.54}$$ 31

This speaks in favor of the complex reaction network of the ongoing catalyst cycle in the selective glycerol oxidation, where the partial reactions do not have integer reaction orders.

### Activation Energy

To better understand the homogeneous glycerol oxidation catalysis, the activation energy is an important parameter for categorizing the implemented reaction and comparing it to other reactions. Analogue to the process described before, the rate constant *k′* was determined by calculating the initial reaction rate using eq 22. After logarithmizing and linearization the resulting plot is shown in Figure 12, including a linear regression of the five determined values including the standard deviation of the reference experiments.

**Figure 12:**
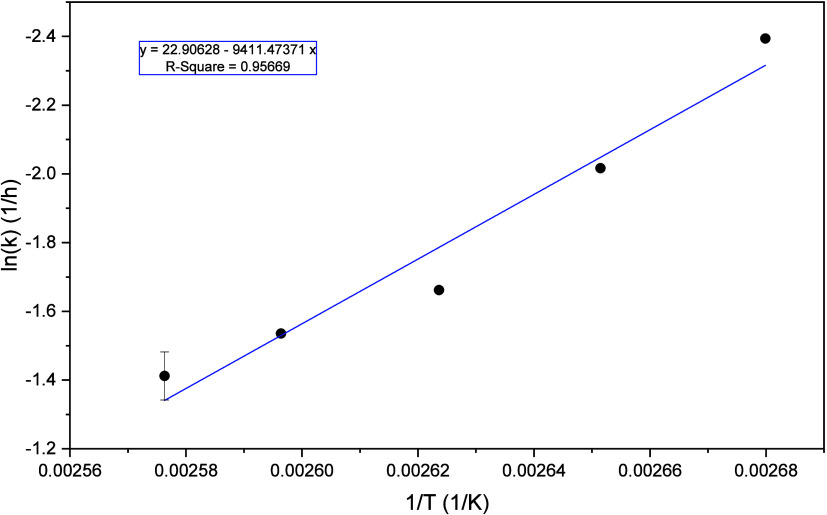
Arrhenius plot: plot of the natural logarithm of the rate constants from the experiments from 100 to 115 °C against the reciprocal temperature with linear regression through all values. (*V̇*_g_ = 650 $\frac{\mathrm{NmL}}{\min}$ , *V̇*_l_ = 1729 $\frac{\mathrm{mL}}{\min}$ , 5 bar_Oxygen_, *c*_Substrate_ = 10 wt % glycerol, *c*_Catalyst_ = 5 $\frac{\mathrm{mmol}}{L}$ HPA-2, *t* = 6 h).

The slope of the linear regression is equal to $-\frac{E_{A}}{R}$ , so multiplying by the general gas constant R yields the activation energy. This results in an activation energy of 78.3 kJ/mol for the glycerol oxidation reaction, which aligns with an earlier study by Voß et al.^41^ on the OxFA process. That study was conducted in STR and determined an activation energy of 83.2 kJ/mol. Moreover, most activation energies in solution are in the range of 30 to 100 kJ/mol.^63^ Furthermore, the selective oxidation of glycerol is an exothermic reaction, where an increase in temperature leads to an increase in reaction rate. With the activation energy determined, it can be assumed that the reaction is in the typical kinetically limited range, since the kinetic energy barrier lies in the typical range for this type of limitation.^63^

### Stability of the HPA-2 Catalyst over Multiple Reactions

In terms of industrial applications, it is important to determine the stability of the catalyst over multiple reactions. To investigate the structure of HPA-2 and the oxidation state of the catalytically active vanadium in HPA-2, a long-term experiment was conducted. The HPA-2 catalyst was used in fed-batch mode for three consecutive runs, with a reaction time of 6 h each. At the start of each test run, fresh glycerol was added to the reactor to achieve the same concentration as at the start of run 1, resulting in initial concentrations of 0.495 mol/L on run 1, 0.428 mol/L on run 2 and 0.460 mol/L on run 3. ^51^V-NMR spectra were recorded both at the start of each test run and after 6 h of operation, the results can be seen in Figure 13 alongside the corresponding pH values. The ^51^V-NMR spectra were calibrated according to the HPA-1 reference (−533.6 ppm). According to Petterson et al.,^64^ the chemical shift of HPA-1 is independent of the pH value.

**Figure 13:**
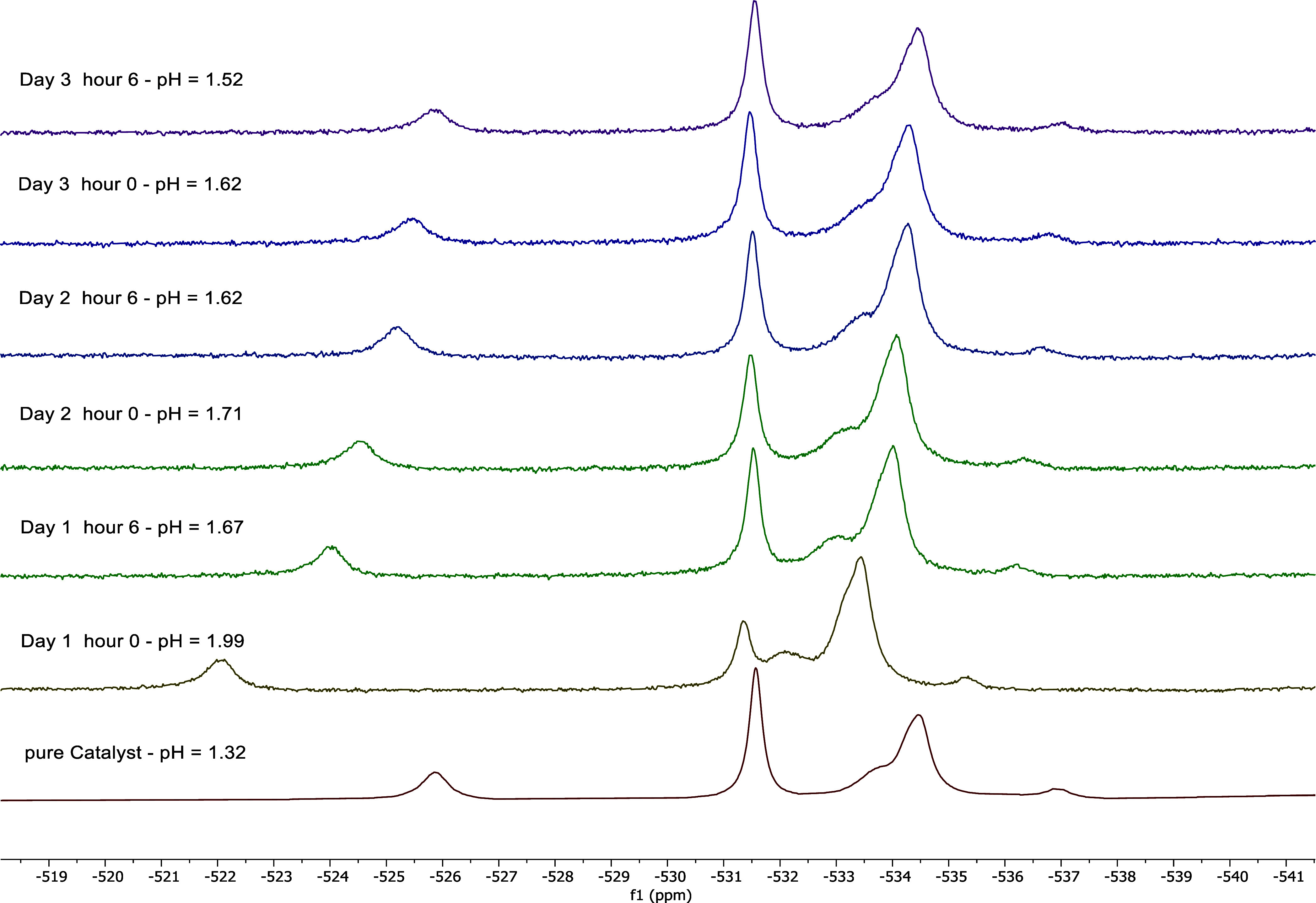
^51^V-NMR spectra stacked over the reaction time for the experiments with 0.495 mol/L glycerol for run 1, 0.428 mol/L glycerol for run 2 and 0.460 mol/L glycerol for run 3 including time of sample removal and pH value of the sample (*V̇*_g_ = 650 $\frac{\mathrm{nmL}}{\min}$ , *V̇*_l_ = 1729 $\frac{\mathrm{mL}}{\min}$ , 5 bar_Oxygen_, 115 °C, 5 wt % glycerol, 5 $\frac{\mathrm{mmol}}{L}$ HPA-2, 3 days each 6 h).

The pH value decreases due to the formation of FA during the course of the reaction, which can also be seen in the measured pH values in Figure 13, where the pH drops down to a value of 1.52 after run 3. According to Petterson et al.^64^ the change in the chemical shift is particularly significant in the range below a pH value of 2. At pH values above 2, the signals do not change and a plateau is reached. This is consistent with the measured ^51^V-NMR spectra. The α-1,4 isomer is in the range from −521 to −528 ppm, the α-1,5; −1,2 and −1,6 isomers are in the range from −534 ppm to −537 ppm, the α-1,11 signal has a low intensity and can be seen at −538 ppm. Consequently, the ^51^V-NMR spectra in Figure 13 confirm the stability of the HPA-2 structure after multiple reactions.^65^

Additionally, it should be noted that vanadium V^IV^ is paramagnetic, meaning that reduced HPA-2 would not be visible in the ^51^V-NMR. This is because the unpaired electron in the d orbitals of V^IV^ interacts with the external magnetic field applied by the NMR device, resulting in strongly broadened and blurred signals. Figure 13 shows that a ^51^V-NMR was detected at each time point, indicating that HPA-2 predominantly exists in an oxidized form with V^V^ after all multiple reactions. This could be further confirmed by UV–vis spectroscopy. The UV–vis spectrum detects the interval charge transfer (IVCT) band of the V–O–Mo group of HPA-2 at 780 nm. In HPA-2, the intensity of the hetero IVCT increases as the catalytically active metal vanadium is reduced from V^V^ to V^IV^. The maximum is reached when the vanadium in HPA-2 is fully reduced. Figure S39 shows the UV–Vis spectra of the long-term test and of pure HPA-2 from 500 to 1000 nm. As can be seen here, the hetero-IVCT bands of the long-term experiment have a lower absorbance than the pure catalyst, which again indicates that HPA-2 is present in its oxidized form after all multiple reactions.

In summary, it can be concluded that the structure and oxidation state of HPA-2 remain stable over multiple reactions.

## Conclusions

This study investigated the selective catalytic oxidation of glycerol to formic acid (FA) comparing the JLR with a conventional STR. Both reactor types exhibited the same product distribution, primarily the selective formation of FA. The JLR demonstrated a superior STY of up to 27.9 g_Fa_ L_R_^–1^ h^–1^ within the first 3 h of reaction time. This outperforms the initial STY of the JLR by a factor of 1.5. This difference is due to the JLR’s excellent mixing properties and high gas input. The convergence of the STY curves of the JLR and STR can be explained by the depletion of glycerol, becoming apparent from the conversion curves. The results highlight the economic advantages of using the JLR for glycerol oxidation.

Additionally, using glycerol – a sustainable byproduct of biodiesel synthesis – is an effective way to produce green FA from renewable resources. To advance in the direction of this goal, both the JLR used and the reaction kinetics of the selective glycerol oxidation to FA were examined. High *k*_l_ · *a* values with a maximum of 173 h^–1^ and a maximum of 8.53 kW ·m^–3^ for the specific energy dissipation rate ε_liquid_ ensure a high gas input into the liquid phase in the JLR. Kinetic investigations revealed no mass transfer limitations in the system using the JLR; the Hatta number of 0.013 in the standard reference experiment indicates that the oxidation is slower than the oxygen mass transfer into the liquid phase. Understanding kinetic parameters is crucial for future scale-up. The reaction orders for oxygen and glycerol were determined to 0.83 and 0.54, respectively. An Arrhenius plot was utilized to calculate the activation energy for glycerol oxidation, resulting in 78.3 kJ/mol, which is within the typical range for kinetically limited reactions of 30–100 kJ/mol. Additionally, the stability and oxidized state of HPA-2 were confirmed over multiple reactions using ^51^V-NMR and UV–vis spectroscopy.

The presented findings lay a solid foundation for the sustainable production of FA with a JLR reactor, which is more efficient in terms of STY and economically feasible than the classic STR. The findings also offer the critical parameters necessary for scaling the process from a pilot plant to a commercial plant setting.

## Supplementary Material



## Data Availability

Research data is available after official publication (DOI: 10.5281/zenodo.17708165).

## Notes

Used materials; description of synthesis of the used HPA-2 catalyst; catalyst characterization, as well as equipment specific descriptions for pump calibration, characterization of the used JLR as well as STR, together with respective HPLC chromatograms and lists of kinetic results can be found in the Supporting Information (PDF)
