# Supplementary material for: Advanced Glycerol Oxidation to Formic Acid in a Multiphasic Jet Loop Reactor Using Polyoxometalate Catalysts
Source: ACS Sustain Chem Eng. 2025 Dec 17;14(1):551–65. doi: 10.1021/acssuschemeng.5c10177 (PMC12801389; doi:10.1021/acssuschemeng.5c10177)
Supplement: Supplementary file 1 [file sc5c10177_si_001.pdf]

Supporting Information for

Advanced glycerol oxidation to formic acid in a multiphasic jet loop reactor using polyoxometalate catalysts

Ira Christina Wirth[1], Daniel Niehaus[1], Dorothea Voß[1], Michael Schlüter[2], Jakob Albert[1]*

[1] Institute of Technical and Macromolecular Chemistry, Universität Hamburg, Bundesstrasse 45, 20146 Hamburg, Germany

[2]Insitute of Multiphase Flows, Technische Universität Hamburg, Eißendorfer Strasse 38, 21073 Hamburg, Germany

Contents

1 Materials, catalyst synthesis and characterization....2
 1.1 Materials 2
 1.2 Synthesis of the HPA-2 catalyst and its characterization 3
2 Characterization of the different catalyst batches....4
3 Pump calibration and reactor volume determination for the JLR....9
4 Characterization of the JLR....10
5 Flow diagram of the stirred tank reactor (STR) 15
6 HPLC Chromatogram 16
 6.1 HPLC Retention times....16
 6.2 HPLC Calibration 17
7 List of kinetic results....22
 7.1 List of the STY results in the JLR and STR....22
 7.2 List of the yields and selectivities in the JLR and STR 25
 7.3 List and diagram of the conversion in the JLR and STR 27
8 Hatta Number....28
9 Diagrams for determining the reaction order of glycerol 29
10 Diagrams for determining the reaction order of oxygen 33
11 Stability of the catalyst – interval charge transfer (IVCT)....35
12 References....37

This supporting information contains 39 figures and 14 tables on 37 pages.

1 Materials, catalyst synthesis and characterization

1.1 Materials

All chemicals were obtained commercially and used as received without further purification, their purities and suppliers used for catalyst synthesis, HPLC calibration and selective oxidation of glycerol are listed in Table S1.

Table S1: List of used chemicals with purity, supplier and area of application.

Chemical	Purity/ Specification	Supplier	Usage
40 V% Oxygen; 60 V% Nitrogen	99.999 %	AIR LIQUIDE	Glycerol oxidation
Nitrogen	99.999 %	LINDE	Glycerol oxidation
Oxygen	99.999 %	WESTFALEN	Glycerol oxidation
Glycerol	99 %	ALFA AESAR	Glycerol oxidation
Sulfuric acid	95 - 97 %	GRÜSSING	HPLC Eluent
Molybdenum oxide	99.5 %	THERMOS SCIENTIFIC	Catalyst synthesis
Phosphoric acid	85 %	GRÜSSINGER	Catalyst synthesis
Vanadium oxide	99.2 %	ALFA AESAR	Catalyst synthesis
Hydrogen peroxide	30 % solution	VWR CHEMICALS	Catalyst synthesis
Formic acid	98 %	GRÜSSING	Calibration HPLC
Acetic acid	99.8 %	VWR CHEMICALS	Calibration HPLC
Hydroxyacetone	95 %	ALFA AESAR	Calibration HPLC
Dihydroxyacetone	100 %	SIGMA-ALDRICH	Calibration HPLC
Glyceraldehyde	100 %	MERCK	Calibration HPLC
Glycolaldehyde	100 %	MERCK	Calibration HPLC
Glycolic acid	99 %	ACROS ORGANICS	Calibration HPLC
Glyoxal	40 % solution	MERCK	Calibration HPLC

1.2 Synthesis of the HPA-2 catalyst and its characterization

The synthesis of HPA-2 ($H_5PV_2Mo_{10}O_{40}$) was carried out according to a self-modified version of the original synthesis by ODYAKOV *et al.*[1] In principle, molybdenum and vanadium precursors were first synthesized separately. The HPA-2 catalyst was obtained by combining the two precursors and subsequent purification. After the synthesis, a red- orange-brown solid was precipitated and analyzed in detail. Therefore, Inductively coupled plasma- optical emission spectroscopy (ICP-OES) using a ARCOS device from the company SPECTRO (Table S2), Thermogravimetric analysis (TGA, Table S3) using a NETZSCH TG 209 F1 220-10-039-K with the software PROTEUS ANALYSIS from NETZSCH and attenuated total reflectance Fourier-transform Infrared (ATR-FT-IR) spectroscopy using a QATRTM-S single-reflection ATR (with a diamond prism) from SHIMADZU were used. Additional to that the obtained HPA-2 catalyst was dissolved in D_2O and analyzed by ^{31}P- and ^{51}V-Nuclear magnetic resonance (NMR) spectroscopy using a BRUNKER AVANCE III HD 600 MHZ device (parameters for the ^{31}P-NMR: 512 scans, 200 sw and for the ^{51}V-NMR: 1000 scans, 800 sw, d_1: 0.2 s. The corresponding characterization data for the three synthesized HPA-2 batches can be found in Figures S1 to S9 and are in consistent with PETTERSON *et al.*[2] and RAABE *et al.*[3], which confirm successful synthesis of HPA-2.

2 Characterization of the different catalyst batches

Table S2: ICP-OES results for the different HPA-2 batches.

Batch number	m_{HPA-2} [mg] in 10 mL demineralized water	Mo content [mg/L]	P content [mg/L]	V content [mg/L]
1	56.9	3071	128	321
2	63.0	3327	121	358
3	57.9	3274	114	363

Table S3: TGA results for the different HPA-2 batches.

Batch number	m_{HPA-2} [mg]	Water content [%]
1	18.0	10.0
2	11.2	13.5
3	13.2	13.0

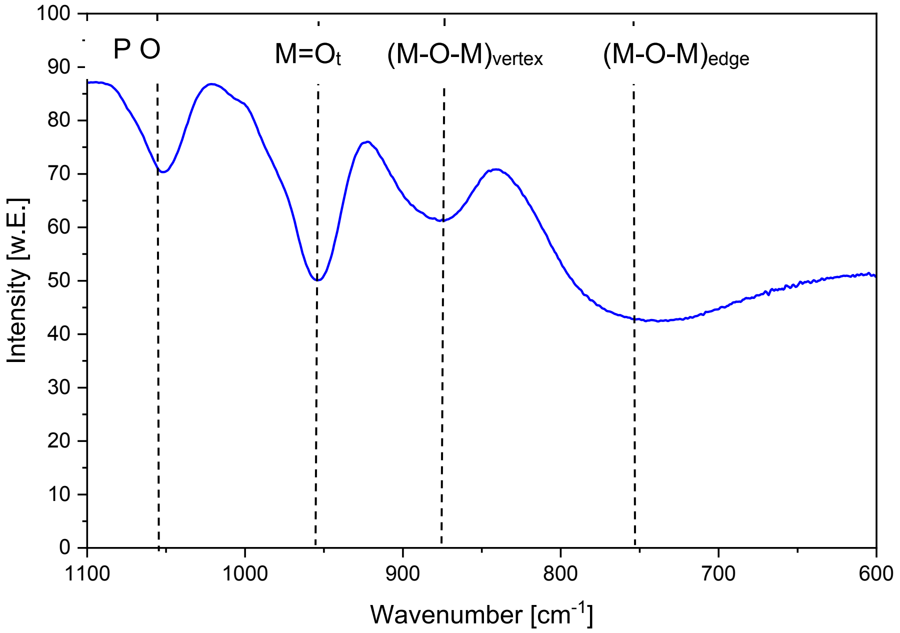

Figure S1: Solid-state ATR-FT-IT spectrum of the HPA-2 (first batch).

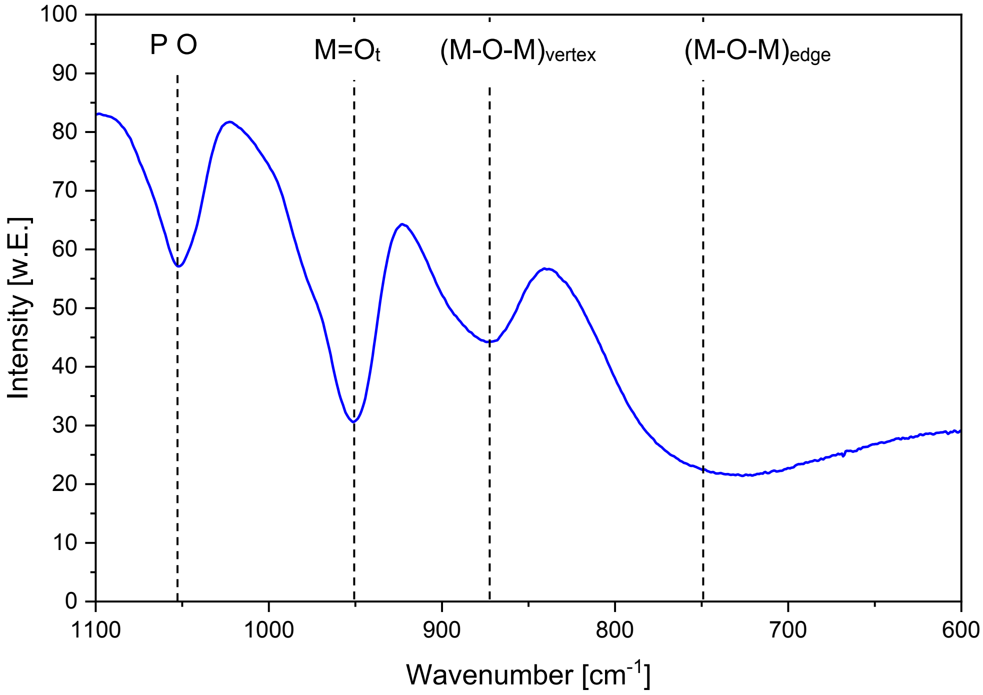

Figure S2: Solid-state ATR-FT-IT spectrum of the HPA-2 (second batch).

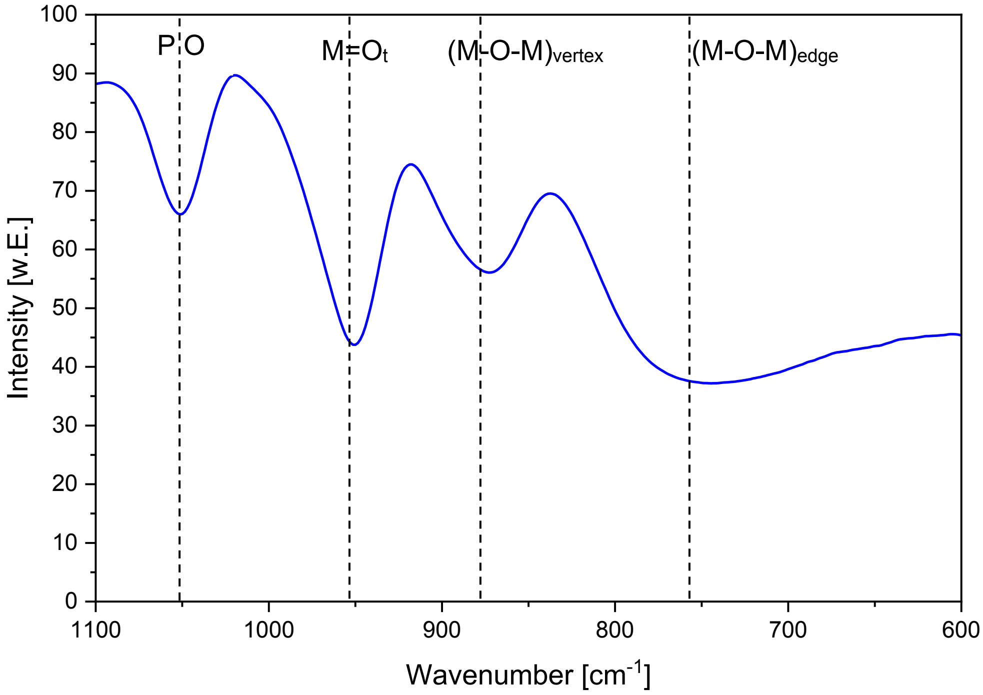

Figure S3: Solid-state ATR-FT-IT spectrum of the HPA-2 (third batch).

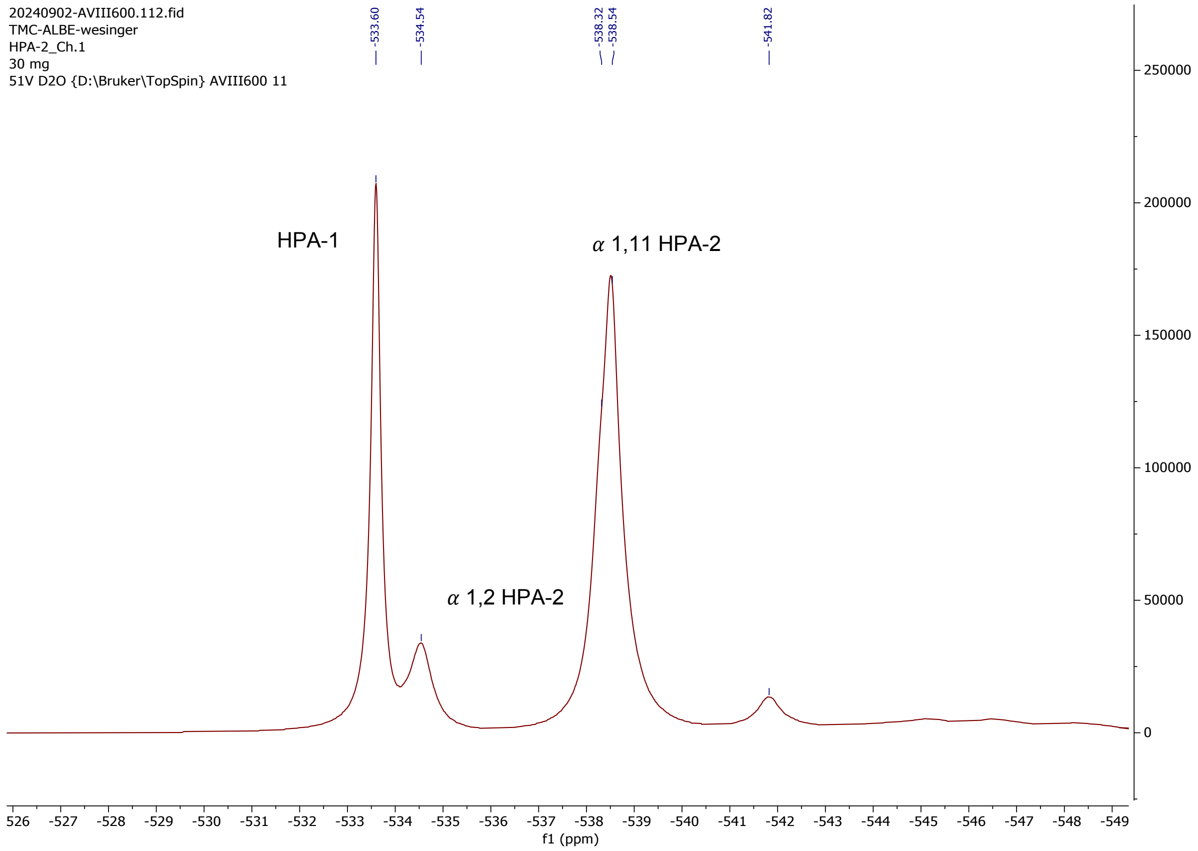

Figure S4: ^{51}V-NMR spectrum of the HPA-2 with 0.1 mL D_2O (first batch, pH = 0.857).

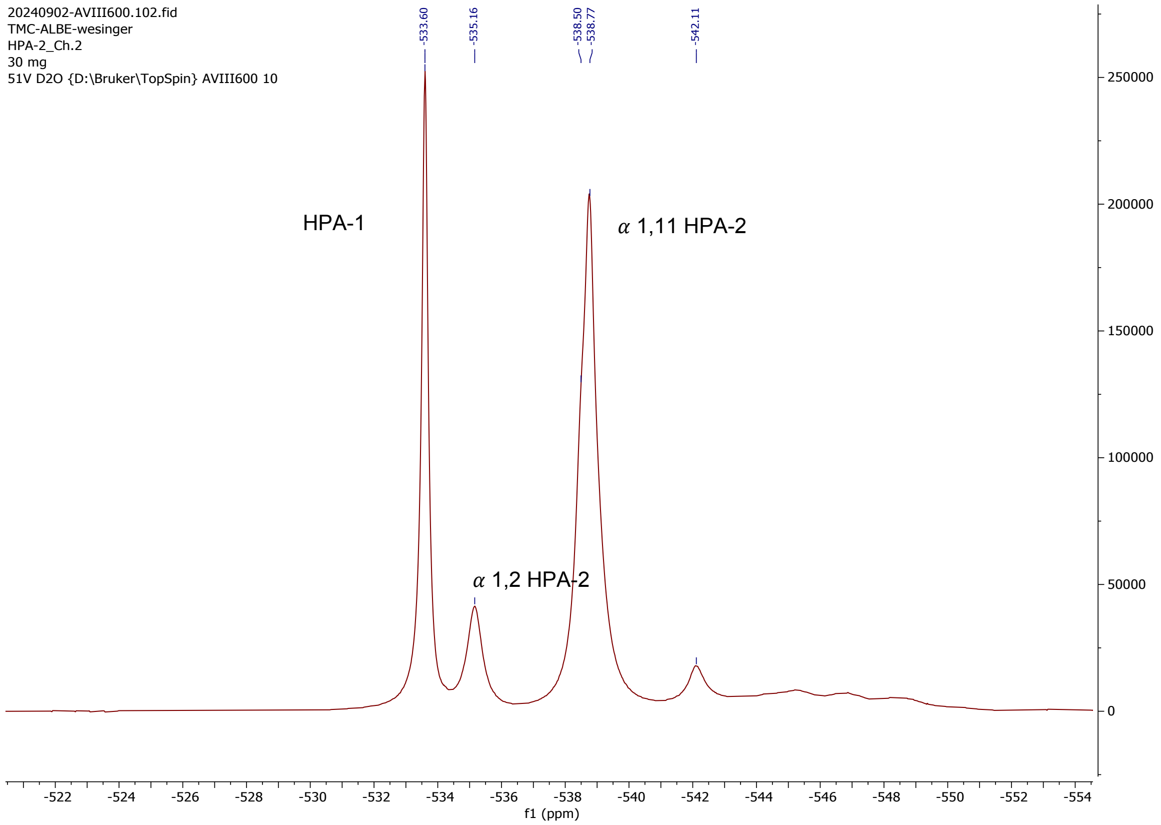

Figure S5: ^{51}V-NMR spectrum of the HPA-2 with 0.1 mL D_2O (second batch pH = 1.28).

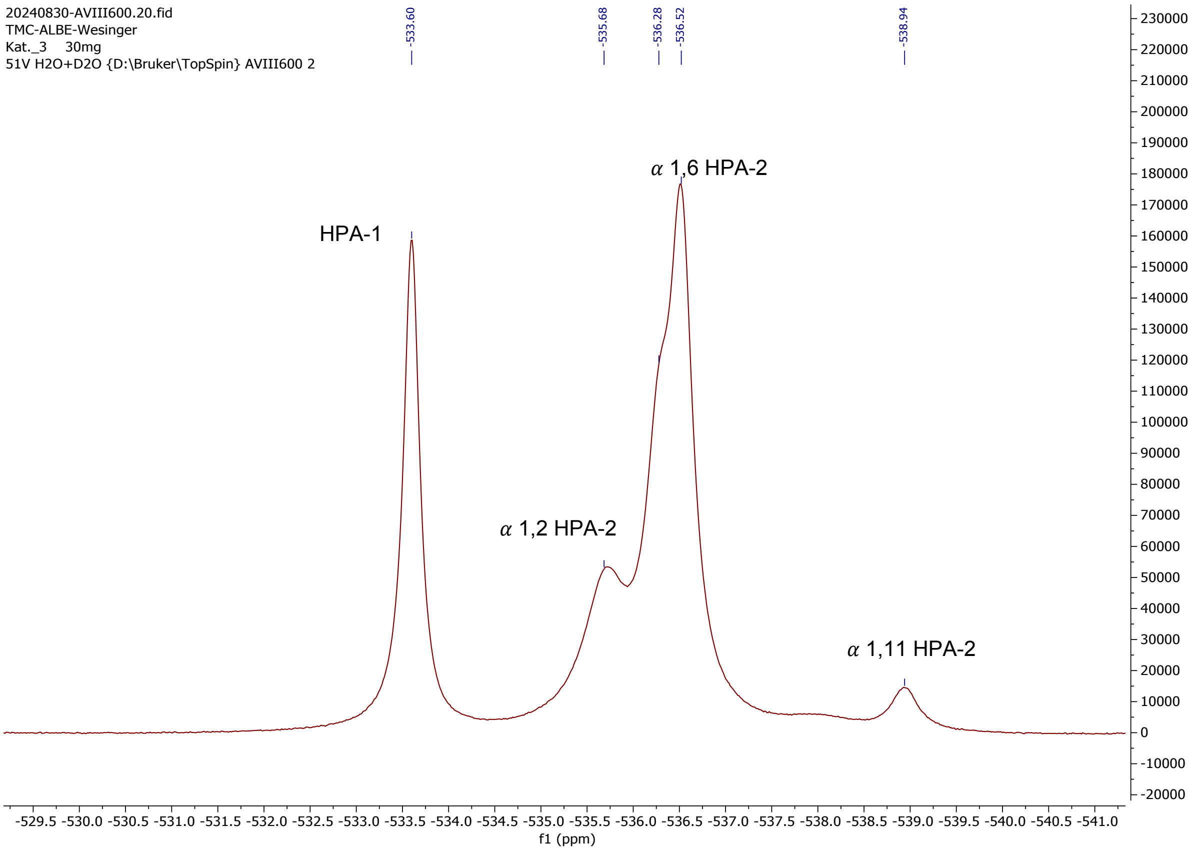

Figure S6: ^{51}V-NMR spectrum of the HPA-2 with 0.1 mL D_2O (third batch pH = 0.847).

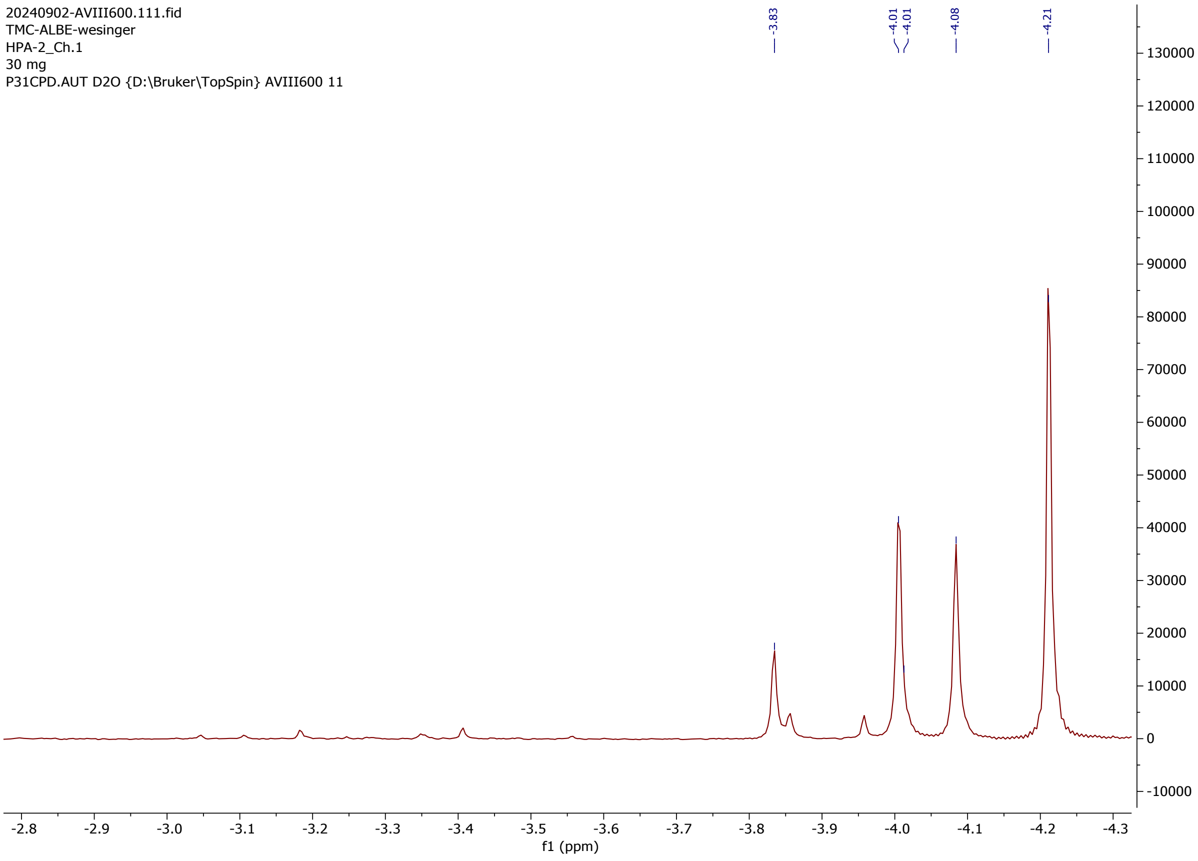

Figure S8: ^{31}P-NMR spectrum of the HPA-2 with 0.1 mL D_2O (first batch pH = 0.857).

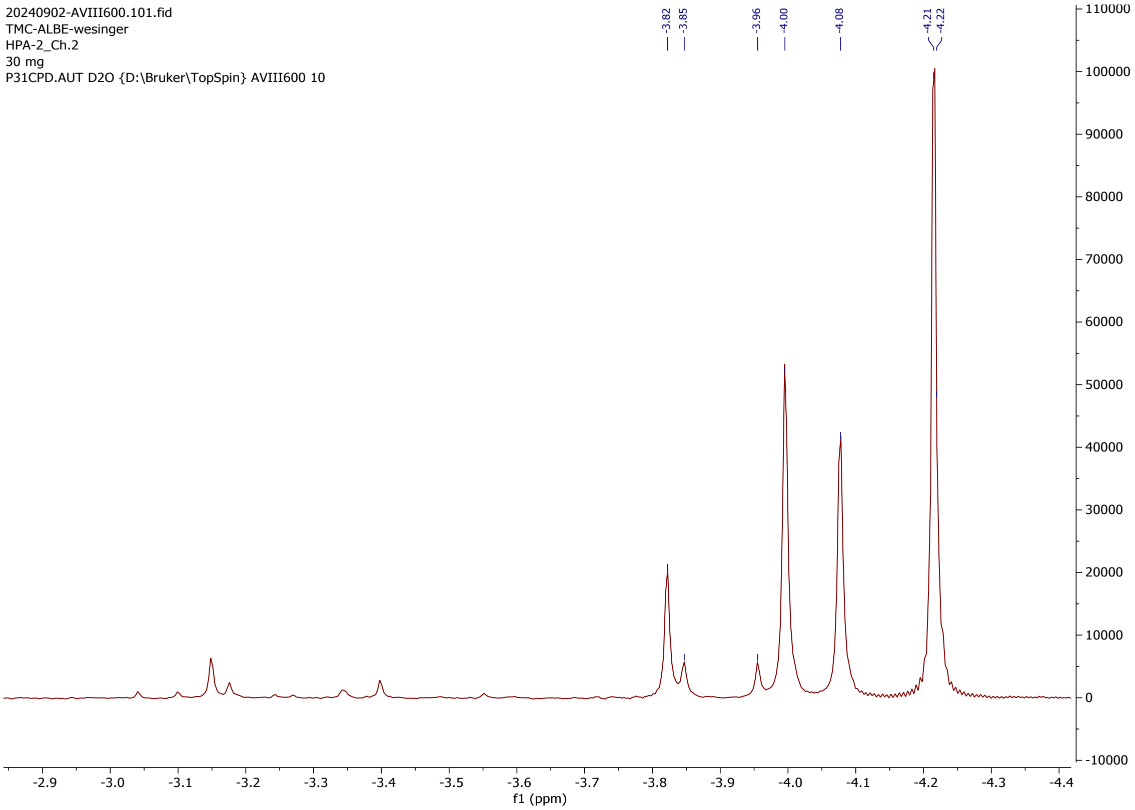

Figure S9: ^{31}P-NMR spectrum of the HPA-2 with 0.1 mL D_2O (second batch pH = 1.28).

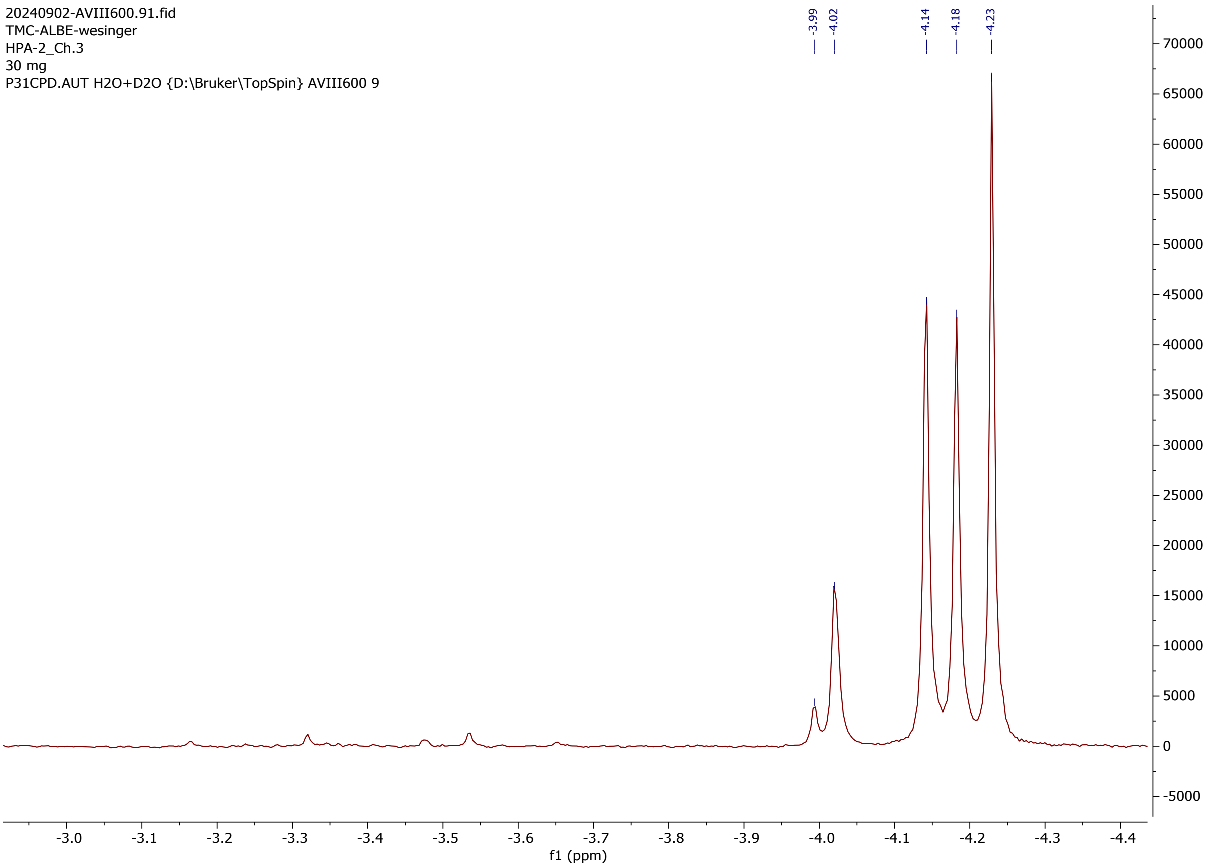

Figure S10: ^{31}P-NMR spectrum of the HPA-2 with 0.1 mL D_2O (third batch pH = 0.847).

3 Pump calibration and reactor volume determination for the JLR

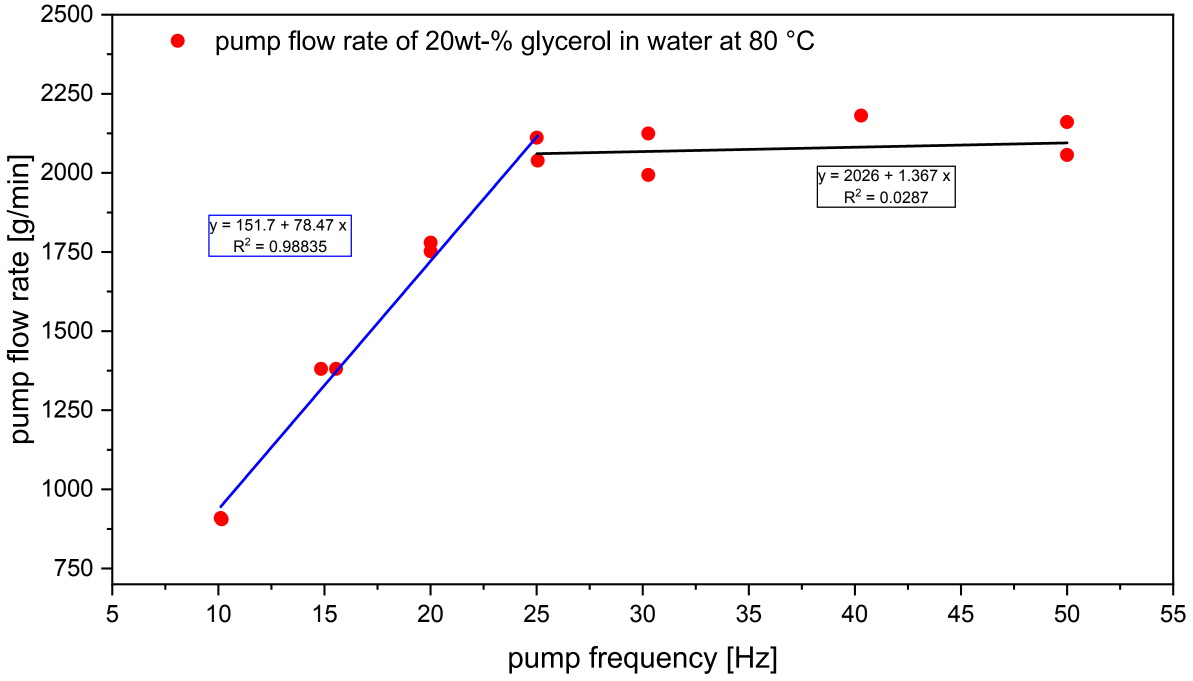

Figure S11: Pump flow rate against the pump frequency with a linear regression (atm, 80 °C, 20 *wt*% glycerol).

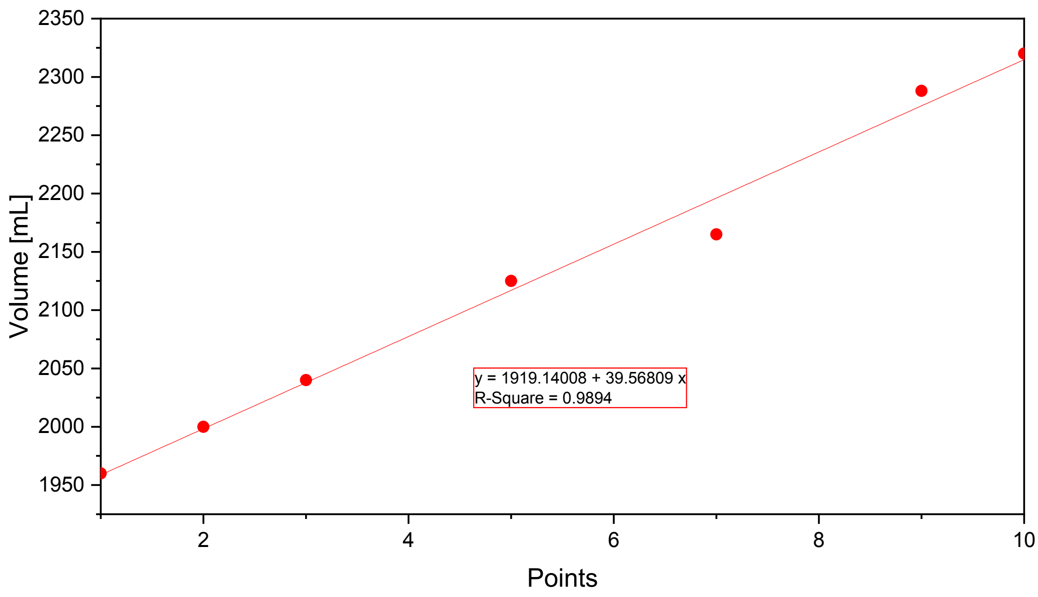

Figure S12: Reactor volume against the points at the upper sight glasses with a linear regression.

4 Characterization of the JLR

Table S4: Nozzle Parameters.

**Diameter of capillary**	0.001587 m
**Capillary Area**	1.978E-06 m^2
**Total nozzle diameter**	0.002 m
**Total nozzle cross-sectional area**	3.142E-06 m^2
**Cross-sectional area A**	1.164E-06 m^2

Table S5: Liquid volume flows and their respective nozzle outlet velocity of the liquid phase and the specific energy dissipation rate.

$\dot{V}_L$ [mL · min^{-1}]	$\omega_{N.liquid}$ [m · s^{-1}]	$\varepsilon_{liquid,\ 80°C}$ [kW · m^{-3}]
1350	19.35	2.21
1729	24.77	4.64
2122	30.40	8.53

Table S6: List of determined values for $k_l \cdot a$ and gas hold-up at different liquid and gas flows. (5 bar_{oxygen}, 80 °C, 20 *wt.*% glycerol)

$\dot{V}_l$ (mL/ min)	$\dot{V}_g$ (mL$_n$,min)	$V_l / \dot{V}_g$	$k_l \cdot a$ (h^{-1})	Gas hold-up (%)
1350	400	4.90	53.8	1.6
1350	400	4.90	52.1	1.6
1349	650	3.02	58.8	3.5
1349	650	3.02	52.96	3.7
1349	150	13.08	24.4	2.9
1349	150	13.08	28.1	2.9
1349	850	2.31	72.8	3.7
1349	850	2.31	66.7	3.7
1728	150	13.08	27.5	2.9
1728	150	13.08	25.5	2.9
1729	400	4.90	49.0	3.1
1728	400	4.90	44.2	3.1

1728	650	3.01	58.0	3.9
1728	650	3.01	57.6	3.7
1728	850	2.30	70.7	3.7
1728	850	2.30	70.8	3.7
2118	150	13.06	25.0	2.4
2118	150	13.06	26.2	2.4
2122	400	4.90	50.8	3.7
2122	400	4.90	50.8	3.7
2122	650	3.01	70.2	3.9
2122	650	3.01	65.9	3.9
2122	850	2.30	81.3	3.9
2122	850	2.30	77.6	3.9

Table S7: List of determined values for $k_l \cdot a$ and gas hold-up at different liquid and gas flows. (5 bar_{oxygen}, 25 °C, demin water)

$\dot{V}_l$ (mL/ min)	$\dot{V}_g$ (mL_n,min)	V_l /$\dot{V}_g$	$k_l \cdot a$ (h^{-1})
1728	150	13.08	51.22
1728	150	13.08	52.86
1729	400	4.90	106.6
1728	400	4.90	108.7
1728	650	3.01	133.8
1728	650	3.01	137.1
1728	850	2.30	159.3
1728	850	2.30	163.3
2000	150	13.08	67.11
2000	150	13.08	67.06
2000	400	4.90	111.2
2000	400	4.90	112.5
2000	650	3.01	145.8
2000	650	3.01	143.8
2000	850	2.30	173.0
2000	850	2.30	169.5

2118	150	13.06	63.61
2118	150	13.06	66.27
2122	400	4.90	117.9
2122	400	4.90	118.4
2122	650	3.01	151.6
2122	650	3.01	152.1
2122	850	2.30	170.6
2122	850	2.30	169.6

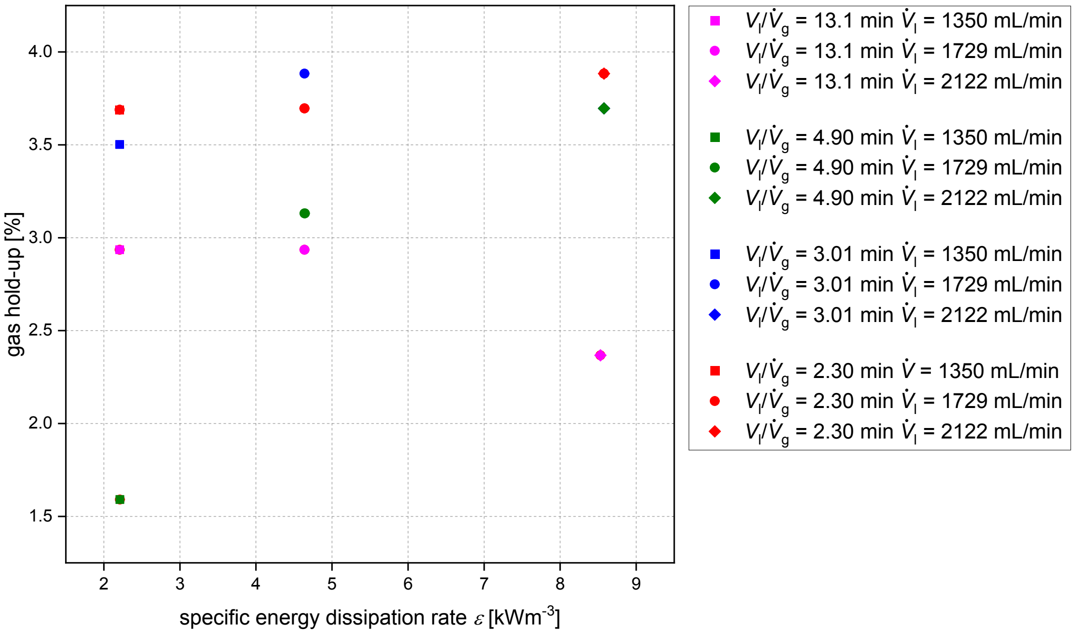

Figure S13: Gas-hold up against the specific energy dissipation rate for different gas- and liquid flows (square: liquid flow $\dot{V}_l$ = 1350 $\frac{mL}{min}$, circle: liquid flow $\dot{V}_l$ = 1729 $\frac{mL}{min}$, rhombus: liquid flow $\dot{V}_l$ = 2122 $\frac{mL}{min}$, 5 bar_{oxygen}, 80 °C, 20 *wt.*% glycerol).

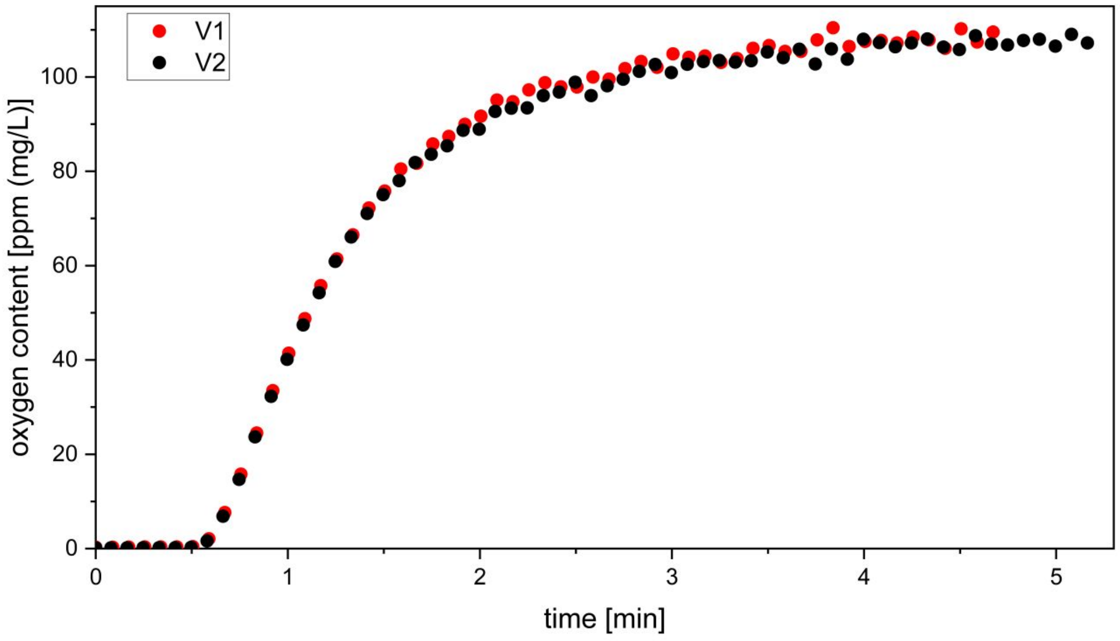

Figure S14: Oxygen content in the liquid phase against the time, double determination of the parameters: 5 bar_{oxygen}, 80 °C, 20 *wt*.% Glycerol, $\dot{V}_l$ = 1729 mL/min, $\dot{V}_g$ = 650 nmL/min.

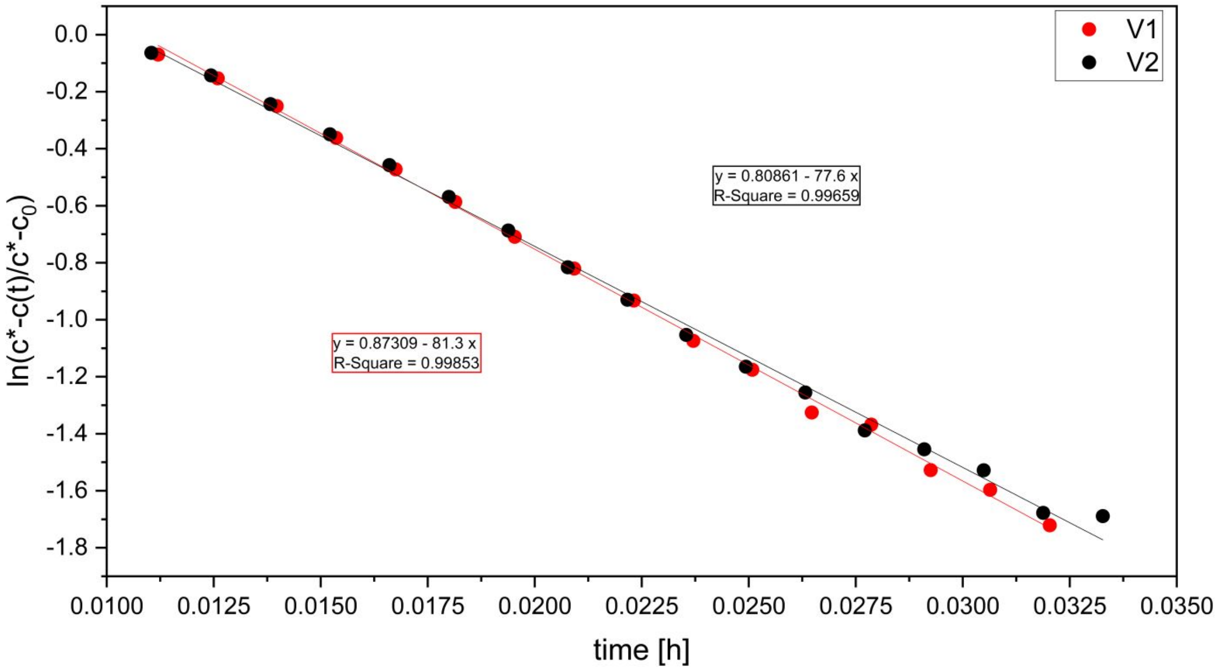

Figure S15: Plot of the natural logarithm of the ratio of the difference between the maximum oxygen concentration and the oxygen concentration at time *t* to the difference between the maximum oxygen concentration and the oxygen concentration at time *t* = 0 h against the time (5 bar_{oxygen}, 80 °C, 20 *wt*.% Glycerol, $\dot{V}_l$ = 1729 mL/min, $\dot{V}_g$ = 650 nmL/min).

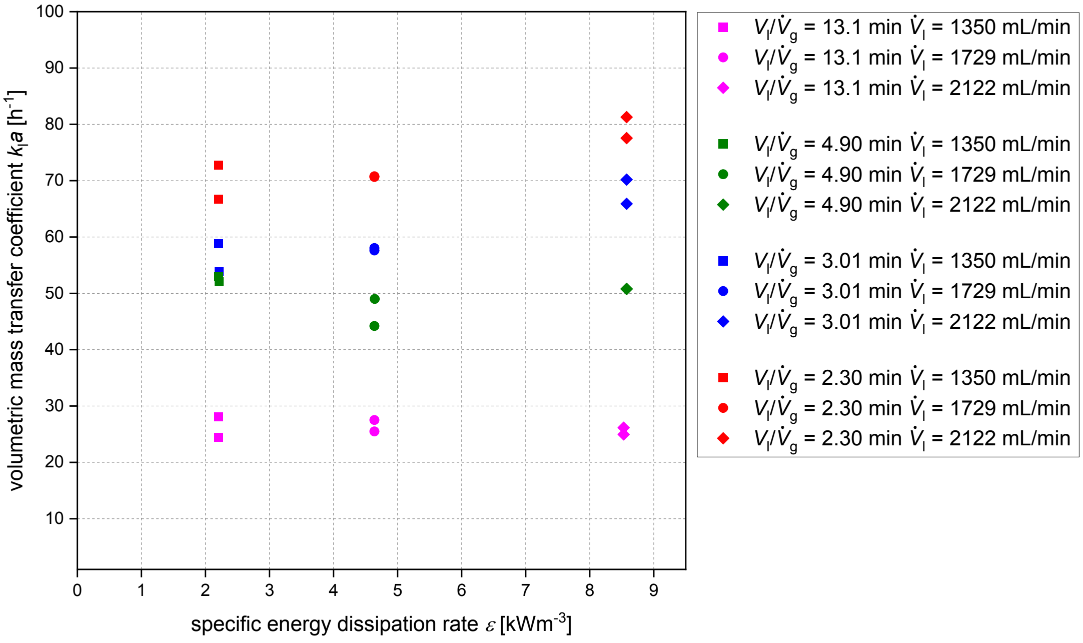

Figure S16: Volumetric mass transfer coefficient plotted over specific energy dissipation rate for different gas- and liquid flows (square: liquid flow $\dot{V}_l$ = 1350 $\frac{mL}{min}$, circle: liquid flow $\dot{V}_l$ = 1729 $\frac{mL}{min}$, rhombus: liquid flow $\dot{V}_l$ = 2122 $\frac{mL}{min}$, p = 5 bar_{Oxygen}, t = 80 °C, 20 wt% Glycerol).

5 Flow diagram of the stirred tank reactor (STR)

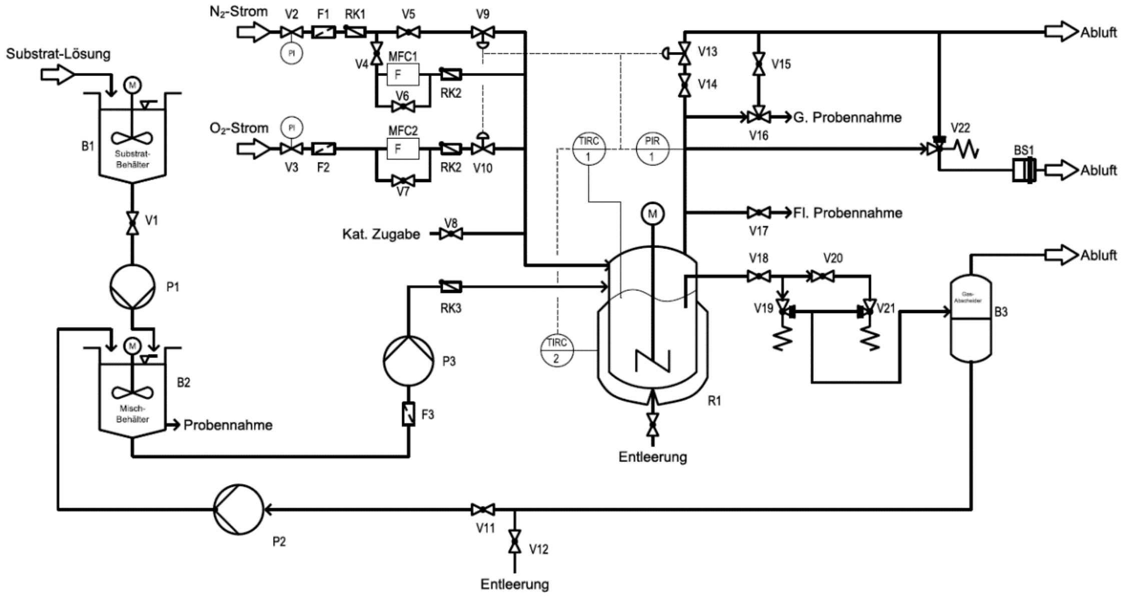

Figure S17: Flow diagram of the fed-batch stirred tank reactor (STR).

6 HPLC Chromatogram

<Chromatogram>

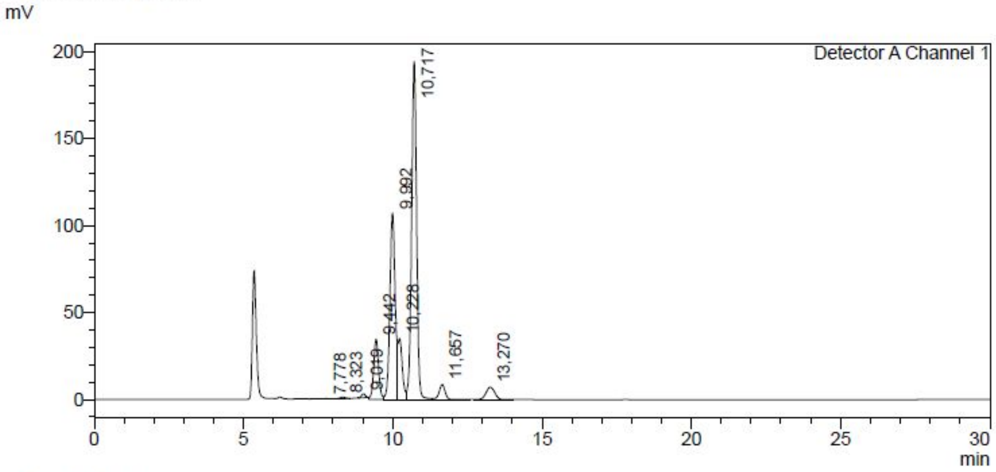

<Peak Table>

Detector A Channel 1

Peak#	Ret. Time	Area	Height	Conc.	Unit	Mark	Name
1	7,778	1103	100	0,001	mol/L	V	Glyoxal
2	8,323	10374	876	0,003	mol/L		Glycerinaldehyd
3	9,019	20160	2148	0,004	mol/L		Glykolaldehyd
4	9,442	398261	34616	0,079	mol/L		Glykolsäure
5	9,992	1336213	106847	0,174	mol/L	V	Glycerin
6	10,228	347749	34905	0,048	mol/L	V	Dihydroxyaceton
7	10,717	2264046	193890	1,275	mol/L	V	Ameisensäure
8	11,657	120507	8619	0,044	mol/L	V	Essigsäure
9	13,270	145550	7138	0,031	mol/L	S	Hydroxyaceton
Total		4643962	389138				

Figure S18: Exemplary HPLC chromatogram of an experiment at 115 °C ($\dot{V}_g$ = 650 $\frac{nmL}{min}$,$\dot{V}_l$ = 1729 $\frac{mL}{min}$, 5 bar$_{Oxygen}$, 112 °C, 10 *wt.*% glycerol, 5 $\frac{mmol}{L}$ HPA-2, 6 h).

6.1 HPLC Retention times

Table S8: List of all calibrated substances and their retention times.

Substance	Retention time [min]
Acetic acid	11.654
Dihydroxy acetone	10.222
Formic acid	10.716
Glyceraldehyde	8.316
Glycerol	9.991
Glycol acid	9.444
Glycolaldehyde	9.016
Glyoxal	7.720
Hydroxy acetone	13.274

6.2 HPLC Calibration

ID# : 1
Name : Glyoxal
Quantitative Method : External Standard
Function : f(x)=3,28961e+006*x-2597,94
Rr1=0,9997723 Rr2=0,9995448 RSS=1,120172e+007
MeanRF: 3,081744e+006 RFSD: 1,618562e+005 RFRSD: 5,252096
FitType : Linear
ZeroThrough : Not Through
Weighted Regression : None
Detector Name : Detector A

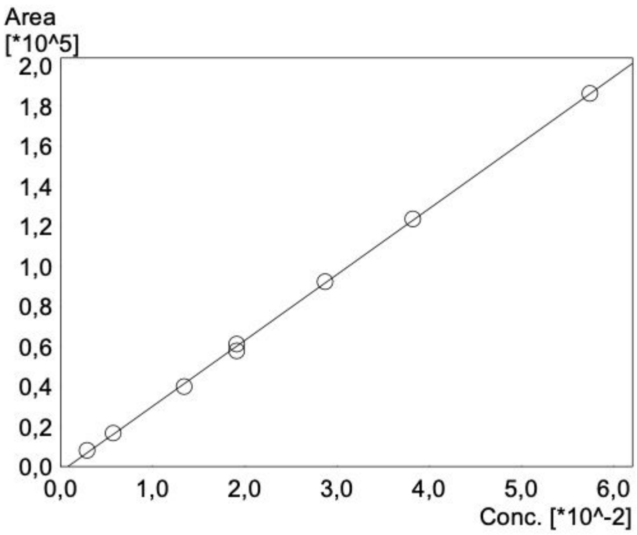

#	Conc.(Ratio)	MeanArea	Area
1	0,0029	8132	8081
			8182
2	0,0057	16713	16640
			16787
3	0,0134	40006	39884
			40129
4	0,0191	57837	57732
			57943
5	0,0191	61245	61152
			61338
6	0,0287	92303	92216
			92391
7	0,0382	123594	123564
			123624
8	0,0574	186319	186271
			186367

ID# : 2
Name : Glykolaldehyd
Quantitative Method : External Standard
Function : f(x)=5,85945e+006*x-2837,52
Rr1=0,9999555 Rr2=0,9999109 RSS=7,522052e+006
MeanRF: 5,622471e+006 RFSD: 2,133184e+005 RFRSD: 3,794032
FitType : Linear
ZeroThrough : Not Through
Weighted Regression : None
Detector Name : Detector A

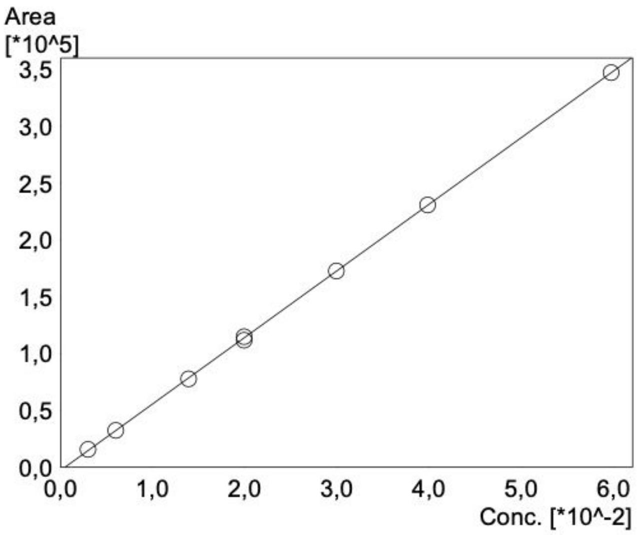

#	Conc.(Ratio)	MeanArea	Area
1	0,003	15591	15531
			15652
2	0,006	32455	32422
			32488
3	0,0139	77881	77846
			77916
4	0,0199	111628	111616
			111640
5	0,0199	114996	114962
			115030
6	0,0299	172630	172627
			172633
7	0,0398	230664	230727
			230601
8	0,0597	347055	347891
			346218

Figure S19: Calibration of glyoxal and glycolaldehyde for the HPLC.

ID# : 3
Name : Glycerin
Quantitative Method : External Standard
Function : f(x)=7,81624e+006*x-22984,9
Rr1=0,9999650 Rr2=0,9999300 RSS=1,263533e+010
MeanRF: 7,737692e+006 RFSD: 1,101359e+005 RFRSD: 1,423368
FitType : Linear
ZeroThrough : Not Through
Weighted Regression : None
Detector Name : Detector A

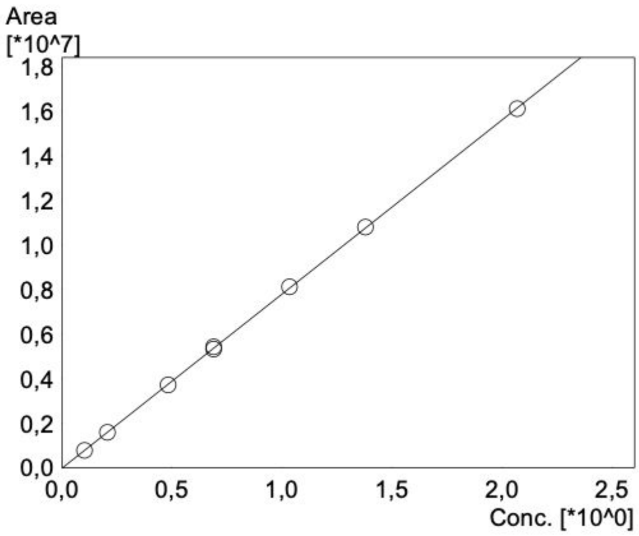

#	Conc.(Ratio)	MeanArea	Area
1	0,1034	779400	776351
			782449
2	0,2069	1578349	1576983
			1579714
3	0,4827	3718743	3717174
			3720313
4	0,6896	5320829	5319952
			5321706
5	0,6896	5420476	5418554
			5422398
6	1,0344	8111936	8111217
			8112655
7	1,3791	10798509	10796732
			10800287
8	2,0687	16100270	16098826
			16101714

ID# : 4
Name : Ameisensäure
Quantitative Method : External Standard
Function : f(x)=1,78565e+006*x-12474,2
Rr1=0,9999598 Rr2=0,9999195 RSS=8,109735e+008
MeanRF: 1,754598e+006 RFSD: 3,159666e+004 RFRSD: 1,800792
FitType : Linear
ZeroThrough : Not Through
Weighted Regression : None
Detector Name : Detector A

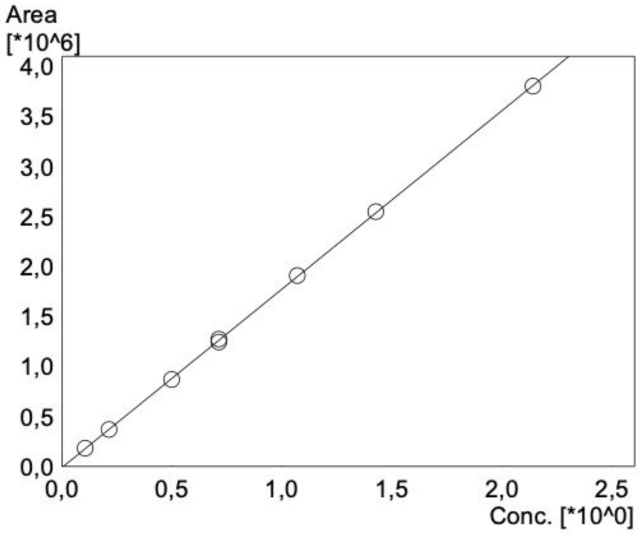

#	Conc.(Ratio)	MeanArea	Area
1	0,107	182021	181327
			182715
2	0,2144	368694	367989
			369399
3	0,4993	870615	870251
			870979
4	0,7133	1243614	1244874
			1242354
5	0,7133	1273982	1273805
			1274158
6	1,07	1907286	1906641
			1907930
7	1,4267	2545451	2547280
			2543622
8	2,14	3800974	3801922
			3800027

Figure S20: Calibration of glycerol and formic acid for the HPLC.

ID# : 5
Name : Hydroxyaceton
Quantitative Method : External Standard
Function : f(x)=4,71723e+006*x-251,613
Rr1=0,9999881 Rr2=0,9999761 RSS=3,002480e+006
MeanRF: 4,723999e+006 RFSD: 9,150779e+004 RFRSD: 1,937083
FitType : Linear
ZeroThrough : Not Through
Weighted Regression : None
Detector Name : Detector A

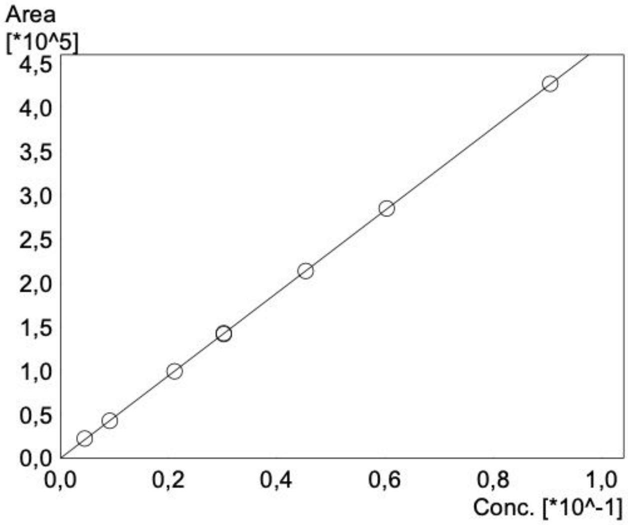

#	Conc.(Ratio)	MeanArea	Area
1	0,0045	22113	22719
			21507
2	0,0091	42409	42477
			42342
3	0,0211	98846	98787
			98904
4	0,0302	141190	141189
			141191
5	0,0302	142284	142304
			142264
6	0,0453	213274	213253
			213295
7	0,0603	284808	284814
			284802
8	0,0905	426720	426705
			426735

ID# : 6
Name : Glycerinaldehyd
Quantitative Method : External Standard
Function : f(x)=5,15963e+006*x-4404,49
Rr1=0,9988777 Rr2=0,9977566 RSS=3,753070e+007
MeanRF: 4,490230e+006 RFSD: 3,847634e+006 RFRSD: 85,689036
FitType : Linear
ZeroThrough : Not Through
Weighted Regression : None
Detector Name : Detector A

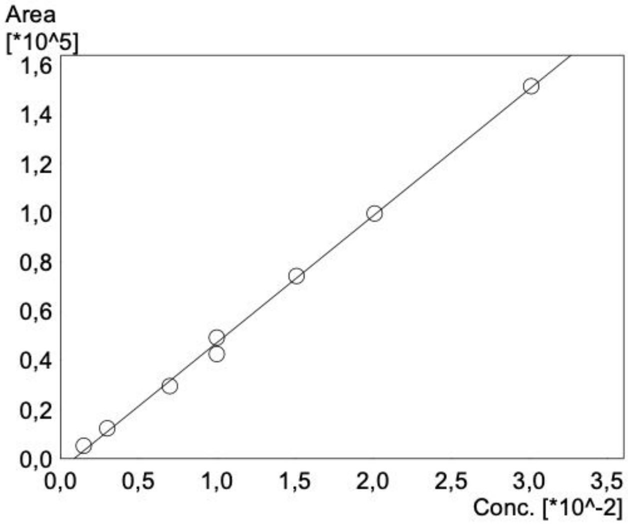

#	Conc.(Ratio)	MeanArea	Area
1	0,0015	5276	1033
			1049
			9480
			9544
2	0,003	12325	2137
			2166
			22533
			22465
3	0,007	29510	5147
			5208
			53830
			53855
4	0,01	42482	7539
			7602
			77450
			77337
5	0,01	49228	7940
			8032
			90269
			90673
6	0,0151	74262	12123

Figure S21: Calibration of hydroxy acetone and glyceraldehyde for the HPLC.

ID# : 7
Name : Glykolsäure
Quantitative Method : External Standard
Function : f(x)=5,18850e+006*x-12976,0
Rr1=0,9993566 Rr2=0,9987136 RSS=5,394241e+008
MeanRF: 4,811382e+006 RFSD: 2,882705e+005 RFRSD: 5,991429
FitType : Linear
ZeroThrough : Not Through
Weighted Regression : None
Detector Name : Detector A

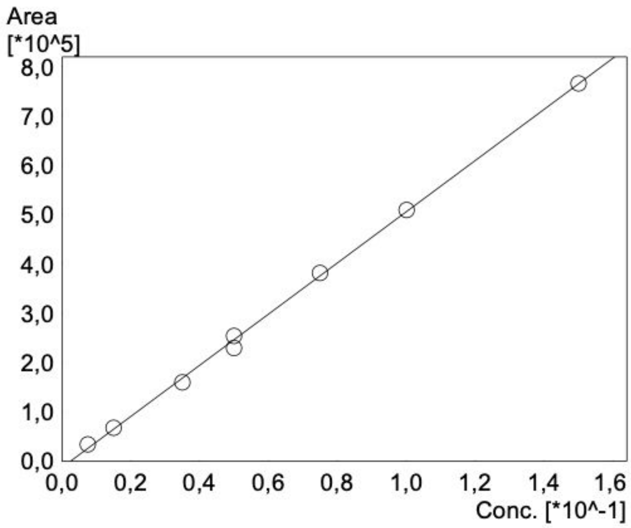

#	Conc.(Ratio)	MeanArea	Area
1	0,0075	33627	33647
			33607
2	0,015	67615	67626
			67605
3	0,035	159626	160064
			159188
4	0,05	229160	229181
			229140
5	0,05	253952	253692
			254213
6	0,075	381304	381389
			381220
7	0,1001	509571	509750
			509392
8	0,1501	765826	765814
			765838

ID# : 8
Name : Dihydroxyaceton
Quantitative Method : External Standard
Function : f(x)=7,71547e+006*x-20069,7
Rr1=0,9993809 Rr2=0,9987621 RSS=1,141172e+009
MeanRF: 7,124551e+006 RFSD: 4,518694e+005 RFRSD: 6,342427
FitType : Linear
ZeroThrough : Not Through
Weighted Regression : None
Detector Name : Detector A

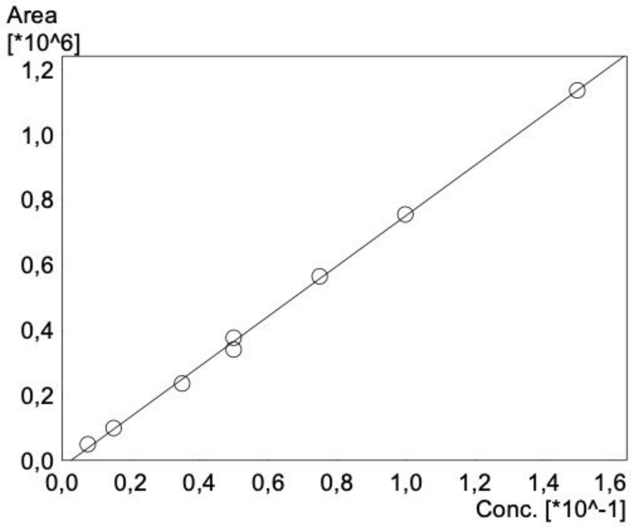

#	Conc.(Ratio)	MeanArea	Area
1	0,0075	49423	49444
			49401
2	0,015	99207	99216
			99197
3	0,0349	235503	237566
			233440
4	0,0499	340337	340407
			340267
5	0,0499	376199	375670
			376729
6	0,0749	564694	564822
			564565
7	0,0998	755097	755634
			754561
8	0,1497	1134752	1134894
			1134611

Figure S22: Calibration of hydroxy acetone and glyceraldehyde for the HPLC.

ID# : 9
Name : Essigsäure
Quantitative Method : External Standard
Function : f(x)=2,91596e+006*x-8212,02
Rr1=0,9988643 Rr2=0,9977298 RSS=1,958295e+008
MeanRF: 2,624109e+006 RFSD: 2,156753e+005 RFRSD: 8,218994
FitType : Linear
ZeroThrough : Not Through
Weighted Regression : None
Detector Name : Detector A

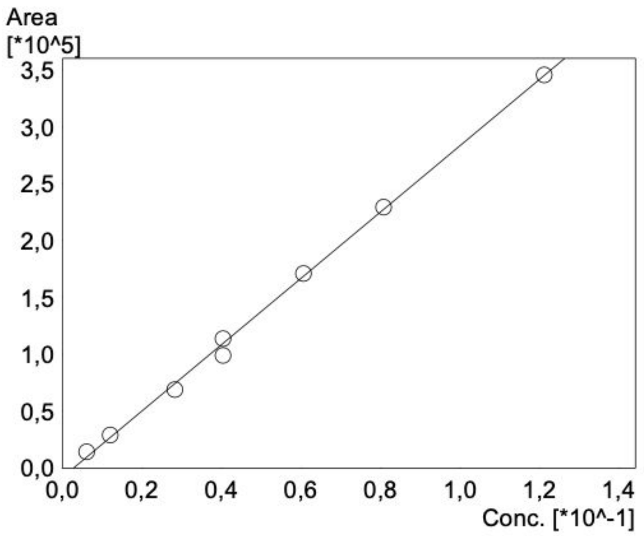

#	Conc.(Ratio)	MeanArea	Area
1	0,0061	14441	14407
			14474
2	0,0121	29170	29177
			29163
3	0,0283	69037	68984
			69090
4	0,0404	98961	98957
			98965
5	0,0404	113711	113569
			113854
6	0,0606	171049	171052
			171046
7	0,0808	229188	229325
			229052
8	0,1211	345389	345325
			345453

Figure S23: Calibration of acetic acid for the HPLC.

7 List of kinetic results

7.1 List of the STY results in the JLR and STR

Table S9: Listing of the STY results at the respective reaction time for three reference experiments in the JLR. *Reaction conditions:* $\dot{V}_g = 650 \frac{NmL}{min}$, $\dot{V}_l = 1729 \frac{mL}{min}$, p = 5 bar_{Oxygen}, T = 115 °C, C_0 = 10 *wt.*% glycerol, $c_{cat} = 5 \frac{mmol}{L}$ HPA-2, t = 6 h.

Time [h]	STY [%] V20_1<br>1.10 mol/L Glycerol	Time [h]	STY [%] V20_2<br>1.06 mol/L Glycerol	Time [h]	STY [%] V20_3<br>1.05 mol/L Glycerol
0.5	27.6	0.5	25.1	0.25	32.4
1	25.1	1	22.2	0.75	23.5
1.5	23.8	1.5	22.4	1.25	23.3
2	22.9	2	21.8	1.75	23.1
2.5	20.7	2.5	19.6	2.25	20.9
3	18.6	3	17.7	2.75	18.8
3.5	16.8	3.5	16.1	3.25	17.0
4	15.2	4	14.6	3.75	15.4
				4.25	14.0

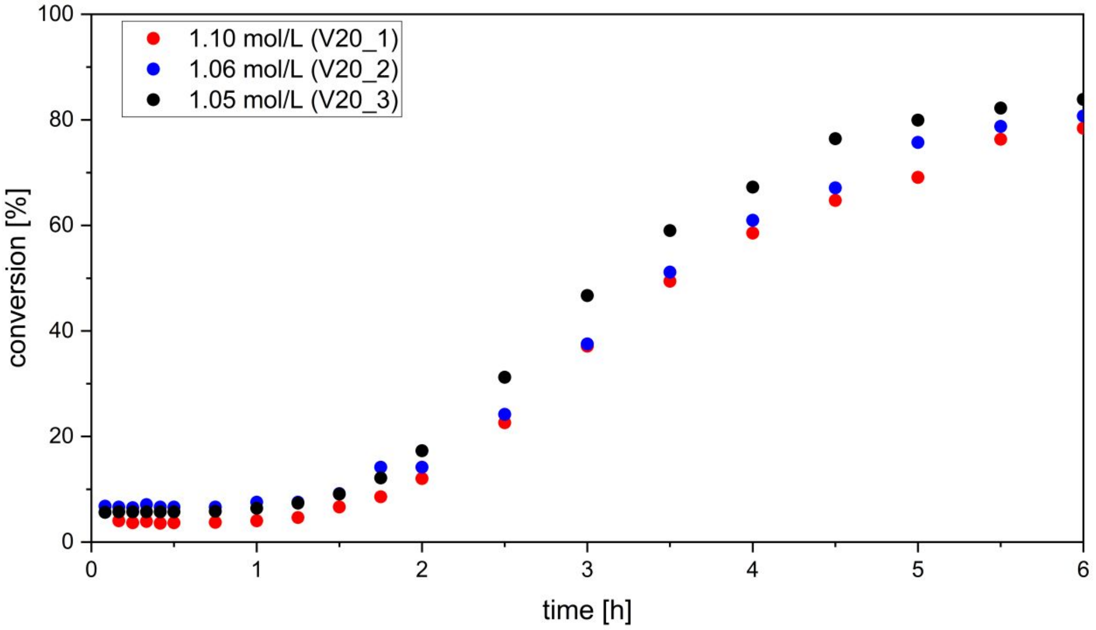

Figure S24: Conversion versus reaction time for the three reference experiments in the JLR (Reaction conditions see Table S8).

Table S10: Listing of the STY results at the respective reaction time for averaged experiments in the STR compared to the average results from Table S8 in the JLR. *Reaction conditions:* $\dot{V}_g$ = 650 $\frac{NmL}{min}$, stirrer speed = 1800 rpm or 1200 rpm, p = 5 bar_{Oxygen}, T = 115 °C, C_0 = 10 *wt.*% glycerol, c_{cat} = 5 $\frac{mmol}{L}$ HPA-2, t = 6 h.

Time [h]	STY [%] STR_1/ 1200 rpm 1.11 mol/L Glycerol	Time [h]	STY [%]STR_2/ 1800 rpm 1.14 mol/L Glycerol	Time [h]	STY [%] V20_ave 1.07 mol/L Glycerol	standard deviation of V20_ave 1.07 mol/L Glycerol
0.083	19.3	0.083	5.52	0.5	26.87	1.24
0.167	11.9	0.166	8.56	1	23.55	1.19
0.25	9.8	0.25	4.71	1.5	23.13	0.57
0.333	8.8	0.333	6.35	2	22.24	0.49
0.583	8.9	0.416	6.19	2.5	20.06	0.49
0.833	11.1	0.5	6.26	3	18.07	0.35
1.083	14.2	0.75	7.73	3.5	16.35	0.31
1.33	18.8	1	10.72	4	14.84	0.25
1.83	18.1	1.25	13.99			
2.333	18.1	1.5	18.50			
2.83	17.1	2	18.90			
3.33	15.9	2.5	18.60			
3.83	14.8	3	17.43			
4.33	13.8	3.5	16.14			
4.83	12.8	4	14.93			
5.33	11.9	4.5	13.80			
		5	12.83			
		5.5	11.94			

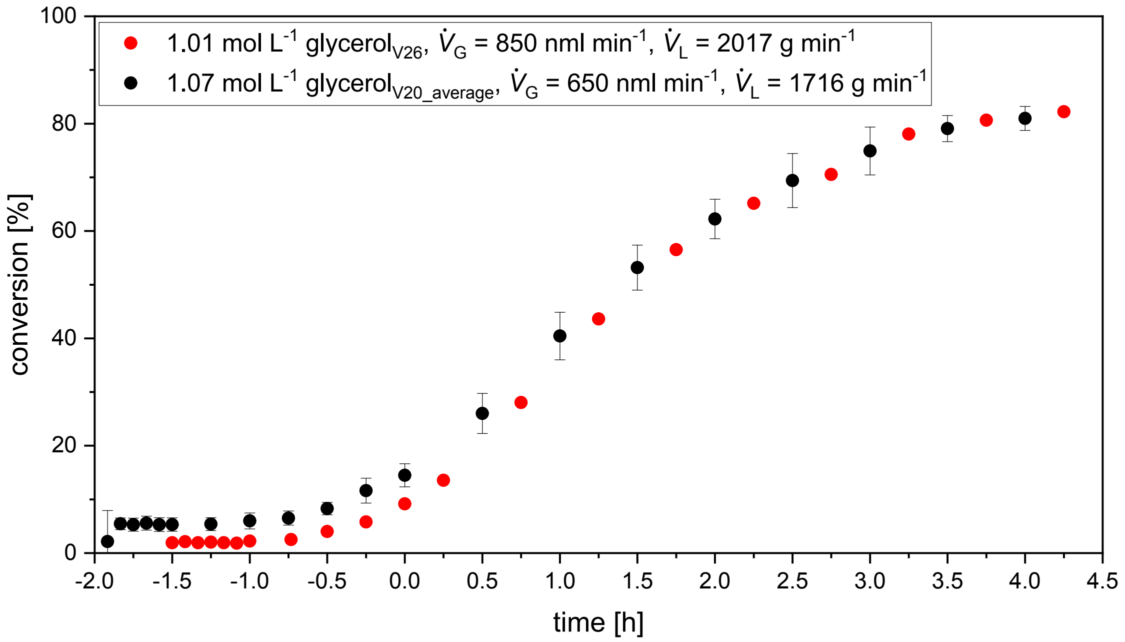

Figure S25*:* Plot of the averaged conversion of the three reference experiments in the JLR including standard deviation against reaction time (*Reaction conditions:* $\dot{V}_g = 650\,\frac{\text{NmL}}{\text{min}}$, $\dot{V}_l = 1729\,\frac{\text{mL}}{\text{min}}$, p = 5 bar_{Oxygen}, T = 115 °C, C_0 = 10 *wt.*% glycerol, $c_{cat} = 5\,\frac{\text{mmol}}{\text{L}}$ HPA-2, t = 6 h.)*,* and plot of the conversion of a higher energy input experiment against reaction time in the JLR. ($\dot{V}_g = 850\,\frac{\text{nmL}}{\text{min}}$, $\dot{V}_l =$ *2122* $\frac{\text{mL}}{\text{min}}$, 5 bar_{Oxygen}, 115 °C, 10 *wt.*% Glycerol, 5 $\frac{\text{mmol}}{\text{L}}$ HPA-2, 6 h).

7.2 List of the yields and selectivities in the JLR and STR

Table S11: Listing of the results for yield and selectivity at the respective reaction time for experiments in the JLR ($\dot{V}_g$ = 650 $\frac{nmL}{min}$,$\dot{V}_l$ = 1729 $\frac{mL}{min}$, 5 bar_{Oxygen}, 115 °C, 10 *wt.*% glycerol, 5 $\frac{mmol}{L}$ HPA-2, 6 h).

**V20_1 1.10 mol/L Glycerol**	**Time [h]**	**1**	**2**	**3**	**4**	**5**	**6**
Yield [%]	Glyoxal	0	0	0.2431	0.1215	0.1215	0.1215
	Glyceraldehyde	0.1823	1.0027	2.6436	2.0966	0.7292	0.4558
	Glycolaldehyde	0.2431	1.2762	2.9778	1.945	0.6685	0.4862
	Glycolic acid	0	1.09389	3.70708	5.34792	4.3148	3.767
	Dihydroxyacetone	0	0	0	0	4.7402	5.04
	**Formic acid**	**0.3950**	**4.1325**	**16.591**	**30.3251**	**38.8331**	**40.2**
	Acetic acid	0	0.8508	1.8231	1.64084	2.18779	2.1877
	Hydroxyacetone	0.0911	0.4557	2.2789	3.55515	4.37557	4.466
	Carbon monoxide	0.0048	0.044	0.329	0.678	0.8377	0.9091
	Carbon dioxide	0	0.0628	1.1581	4.166	6.6718	8.2959
Selectivity [%]	Glyoxal	0	0	0.6552	0.20768	0.1759	0.155
	Glyceraldehyde	4.5454	8.33	7.125	3.58255	1.05541	0.5813
	Glycolaldehyde	6.0606	10.606	8.026	3.32295	1.23131	0.62
	Glycolic acid	0	9.0909	9.99	9.13811	7.03606	4.806
	Dihydroxyacetone	0	0	0	0	0	6.432
	**Formic acid**	**9.8484**	**34.343**	**44.717**	**51.814**	**53.25**	**51.279**
	Acetic acid	0	7.0707	4.914	2.803	3.16623	2.7906
	Hydroxyacetone	2.273	3.7878	6.1425	6.074	5.93667	5.6976
	Carbon monoxide	0.118	0.36	0.88528	1.1589	1.2133	1.1597
	Carbon dioxide	0	0.5226	3.1215	7.119	9.655	10.589

Table S12: Listing of the results for yield and selectivity at the respective reaction time for experiments in the STR ($\dot{V}_g$ = 650 $\frac{nmL}{min}$, 1800 rpm or 1200 rpm, 5 bar_{Oxygen}, 115 °C, 10 *wt.*% glycerol, 5 $\frac{mmol}{L}$ HPA-2, 6 h).

**STR 1200 RPM 1.11 mol/L Glycerol**	**Time [h]**	**1**	**2**	**3**	**4**	**5**	6
Yield [%]	Glyoxal	0	0.65844	0.83801	0.419	0.11972	0.05986
	Glyceraldehyde	0.6285	3.5915	3.4119	1.52637	0.62851	0.26936
	Glycolaldehyde	0.5986	2.5739	1.49645	0.77815	0.47886	0.29929
	Glycolic acid	0.5986	4.3696	8.08081	8.67939	7.30266	5.56678
	**Formic acid**	**1.9155**	**16.311**	**27.4149**	**34.5380**	**38.8477**	**41.4216**
	Acetic acid	0	0.7782	3.35204	4.24991	4.42948	4.48934
	Hydroxyacetone	0.0898	1.3468	2.33446	2.60382	2.6936	2.60382
Selectivity [%]	Glyoxal	0	1.9311	1.39983	0.57845	0.15225	0.0732
	Glyceraldehyde	8.8889	10.533	5.69929	2.10722	0.79931	0.3294
	Glycolaldehyde	8.4656	7.5488	2.49969	1.07427	0.609	0.366
	Glycolic acid	8.4656	12.815	13.4983	11.9822	9.28728	6.80758
	Formic acid	**27.09**	**47.839**	**45.794**	**47.681**	**49.405**	**50.65422**
	Acetic acid	0	2.2822	5.5993	5.86716	5.63327	5.48998
	Hydroxyacetone	1.27	3.950	3.89951	3.59467	3.42564	3.18419

7.3 List and diagram of the conversion in the JLR and STR

Table S13: Listing of the conversion results at the respective reaction time for averaged experiments in the JLR ($\dot{V}_g$ = 650 $\frac{\text{nmL}}{\text{min}}$,$\dot{V}_l$ = 1729 $\frac{\text{mL}}{\text{min}}$, 5 $\text{bar}_{\text{Oxygen}}$, 115 °C, 10 *wt.*% glycerol, 5 $\frac{\text{mmol}}{\text{L}}$ HPA-2, 6 h) and of experiment in the STR ($\dot{V}_g$ = 650 $\frac{\text{nmL}}{\text{min}}$, 1200 rpm, 5 $\text{bar}_{\text{Oxygen}}$, 115 °C, 10 *wt.*% glycerol, 5 $\frac{\text{mmol}}{\text{L}}$ HPA-2, 6 h).

Time [h]	Conversion [%] STR 1200 RPM 1.11 mol/L Glycerol	Time [h]	Conversion [%] V20_ave 1.07 mol/L Glycerol	Standard deviation of V20_ave 1.07 mol/L Glycerol
-1.50	1.935	-1.917	2.141	5.788
-1.42	2.134	-1.833	5.453	1.082
-1.33	1.935	-1.750	5.300	1.213
-1.25	2.035	-1.666	5.580	1.298
-1.17	1.935	-1.583	5.301	1.286
-1.08	1.836	-1.500	5.331	1.245
-1.00	2.233	-1.250	5.393	1.214
-0.73	2.531	-1.000	5.991	1.478
-0.50	4.020	-0.750	6.522	1.327
-0.25	5.806	-0.500	8.299	1.163
0.00	9.181	-0.250	11.625	2.317
0.25	13.548	0.000	14.499	2.157
0.75	28.040	0.500	26.012	3.748
1.25	43.623	1.000	40.443	4.431
1.75	56.526	1.500	53.189	4.187
2.25	65.161	2.000	62.242	3.671
2.75	70.521	2.500	69.413	5.042
3.25	78.065	3.000	74.916	4.463
3.75	80.645	3.500	79.089	2.436
4.25	82.233	4.000	80.991	2.239

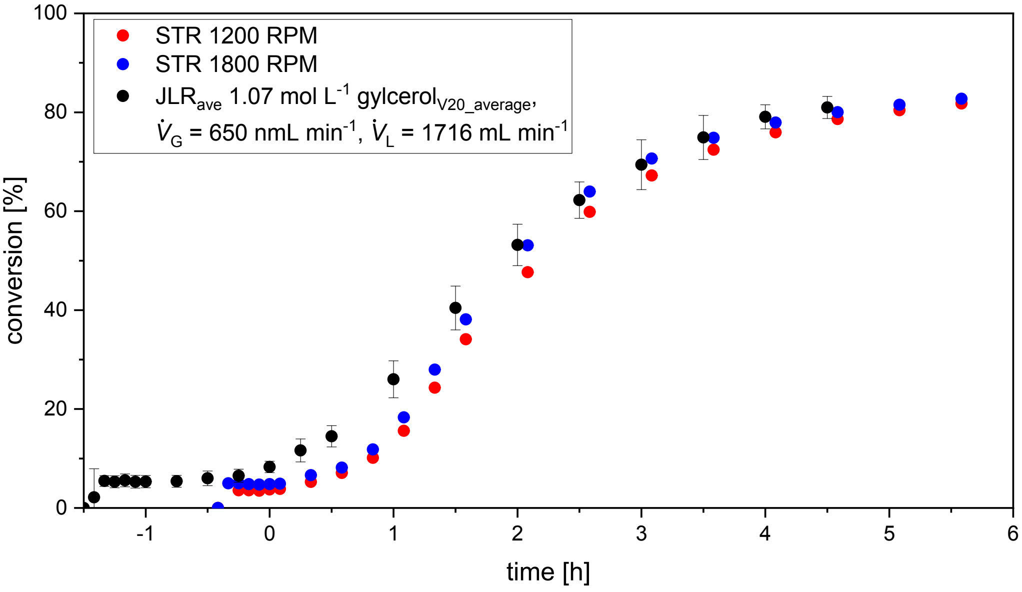

Figure S26: *Average of the conversion (black) versus reaction time for the three reference experiments in the JLR including standard deviation* ($\dot{V}_g$ = *650* $\frac{NmL}{min}$,$\dot{V}_l$ = *1729* $\frac{mL}{min}$, p_{O_2} = 5 bar, *t = 115 °C,* c_0 *= 10 wt.% Glycerol,* c_{Cat} = *5* $\frac{mmol}{L}$ *HPA-2, t = 6 h). In red and blue the conversion for the two experiments in the stirred tank reactor (*$\dot{V}_g$ *= 650* $\frac{NmL}{min}$, *stirrer speed = 1200 rpm or 1800 rpm,* p_{O_2} = 5 bar, *t = 115 °C,* c_0 *= 10 wt.% Glycerol,* c_{Cat} *= 5* $\frac{mmol}{L}$ *HPA-2, t = 6 h).*

8 Hatta Number

Table S14: Hatta number calculated for the averaged experiments with these reaction conditions: $\dot{V}_g$ = 650 $\frac{nmL}{min}$,$\dot{V}_l$ = 1729 $\frac{mL}{min}$, 5 bar$_{Oxygen}$, 115 °C, 10 *wt*.% Glycerol (1.10 mol/ L, 1.06 mol/L and 1.05 mol/L Glycerol) 5 $\frac{mmol}{L}$ HPA-2, 6 h, Gas hold-up$_{averaged}$ = 0.0379, $D_{O_2,\,l}$ = 2 · 10^{-9} m^2 s^{-1}[4], d_{mean} = 0.0038 m[5].

a [m]	δ_l [m]	k_{ave} [mol L^{-1} h^{-1}]	**Ha** [-]
59.84	7.451 · 10^{-2}	24.53	0.014

9 Diagrams for determining the reaction order of glycerol

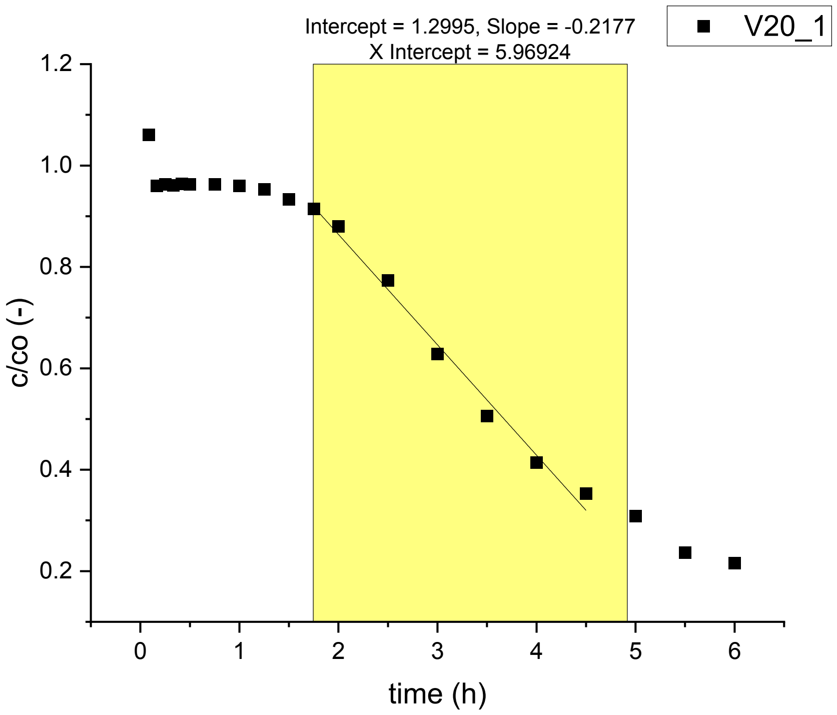

Figure S27: Normalized glycerol concentrations versus the reaction time of the experiment with 1.10 mol/L glycerol including linear regression after the inflection point, point at which the reaction temperature of 115 °C was reached ($\dot{V}_g$ = 650 $\frac{nmL}{min}$,$\dot{V}_l$ = 1729 $\frac{mL}{min}$, 5 bar_{Oxygen}, 115 °C, 5 $\frac{mmol}{L}$ HPA-2, 6 h).

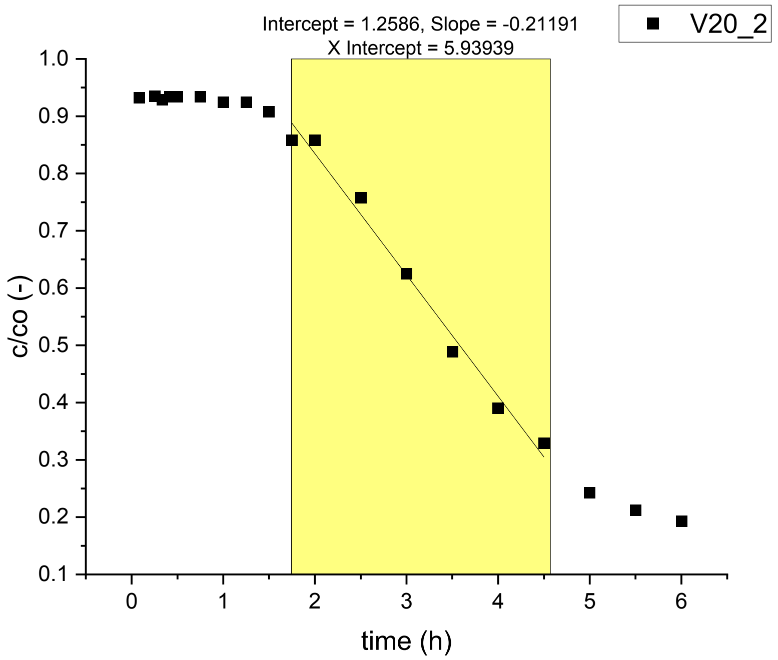

Figure S28: Normalized glycerol concentrations versus the reaction time of the experiment with 1.06 mol/L glycerol including linear regression after the inflection point, point at which the reaction temperature of 115 °C was reached ($\dot{V}_g$ = 650 $\frac{nmL}{min}$,$\dot{V}_l$ = 1729 $\frac{mL}{min}$, 5 bar_{Oxygen}, 115 °C, 5 $\frac{mmol}{L}$ HPA-2, 6 h).

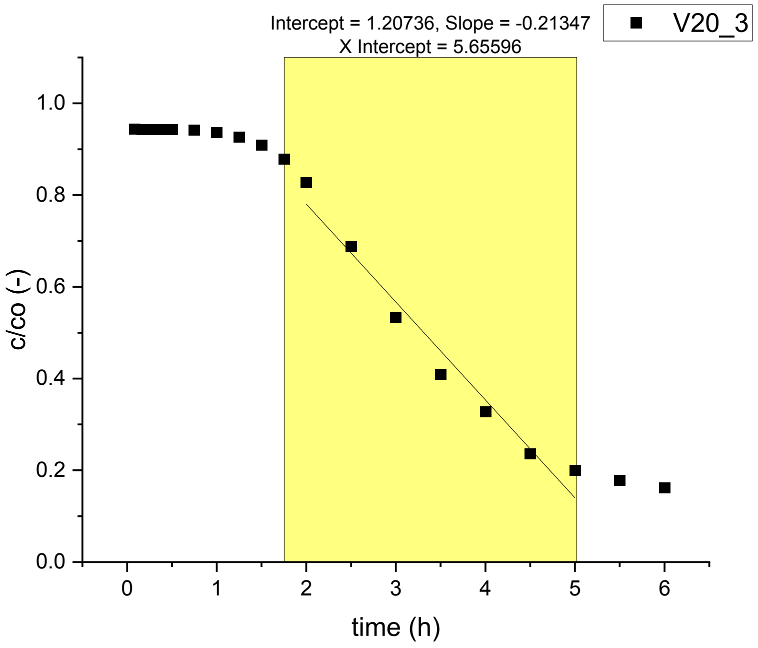

Figure S29: Normalized glycerol concentrations versus the reaction time of the experiment with 1.05 mol/L glycerol including linear regression after the inflection point, point at which the reaction temperature of 115 °C was reached ($\dot{V}_g$ = 650 $\frac{nmL}{min}$,$\dot{V}_l$ = 1729 $\frac{mL}{min}$, 5 bar_{Oxygen}, 115 °C, 5 $\frac{mmol}{L}$ HPA-2, 6 h).

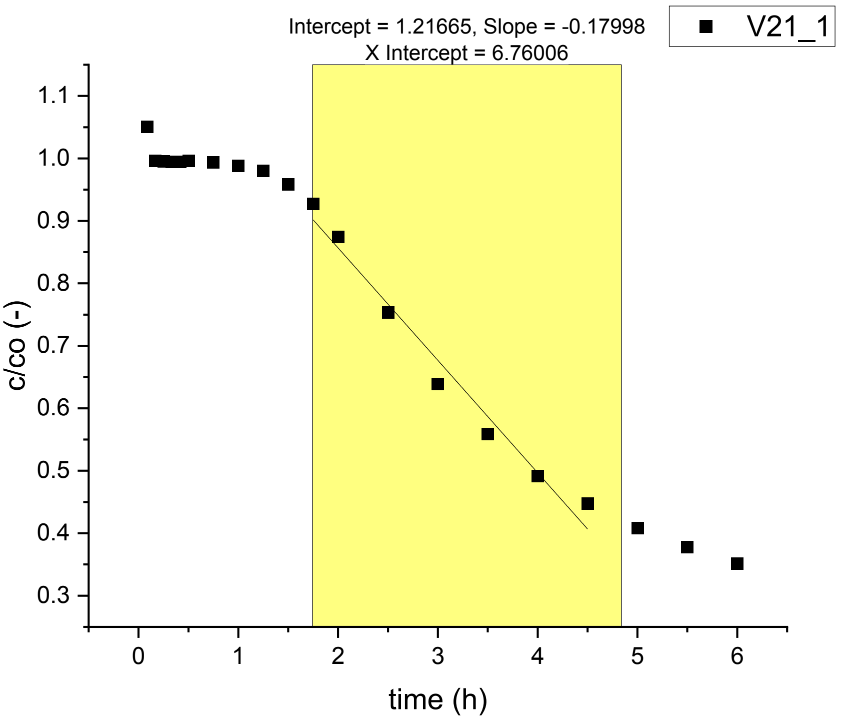

Figure S30: Normalized glycerol concentrations versus the reaction time of the experiment with 2.01 mol/L glycerol including linear regression after the inflection point, point at which the reaction temperature of 115 °C was reached ($\dot{V}_g$ = 650 $\frac{nmL}{min}$,$\dot{V}_l$ = 1729 $\frac{mL}{min}$, 5 bar_{Oxygen}, 115 °C, 5 $\frac{mmol}{L}$ HPA-2, 6 h).

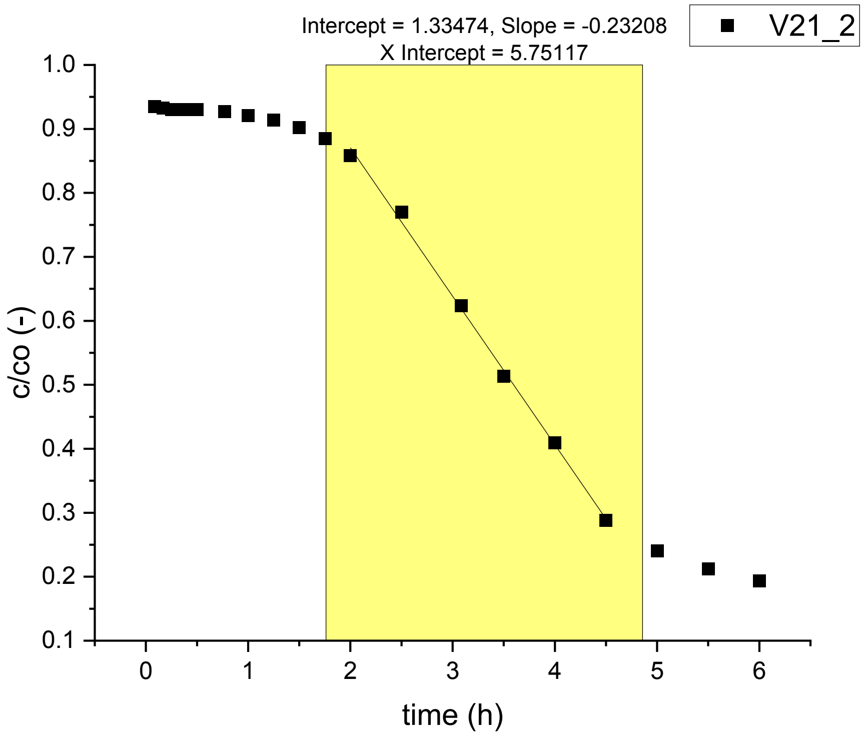

Figure S31: Normalized glycerol concentrations versus the reaction time of the experiment with 0.52 mol/L glycerol including linear regression after the inflection point, point at which the reaction temperature of 115 °C was reached ($\dot{V}_g$ = 650 $\frac{nmL}{min}$,$\dot{V}_l$ = 1729 $\frac{mL}{min}$, 5 bar_{Oxygen}, 115 °C, 5 $\frac{mmol}{L}$ HPA-2, 6 h).

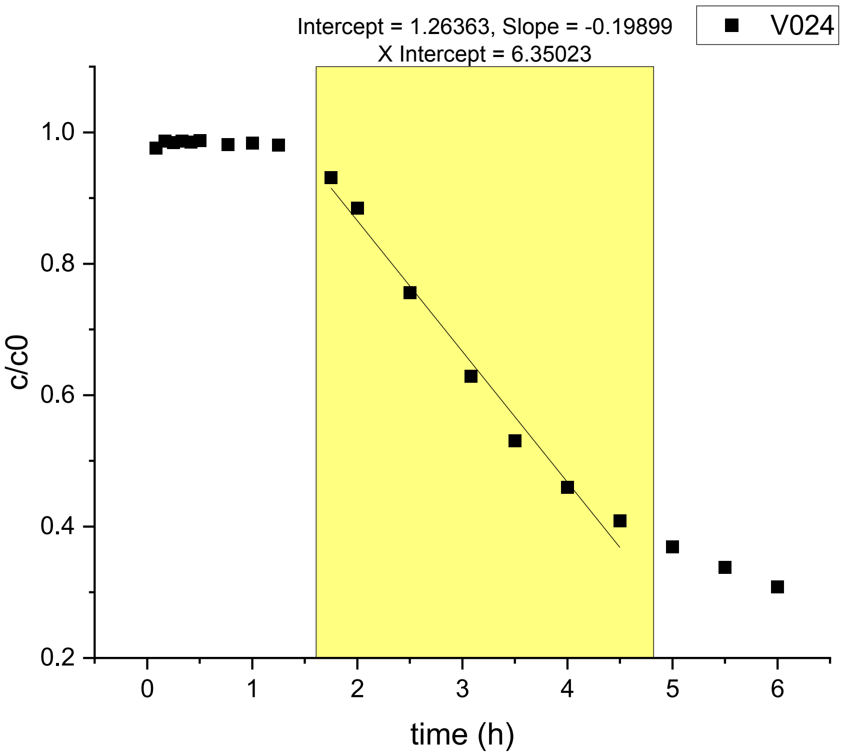

Figure S32: Normalized glycerol concentrations versus the reaction time of the experiment with 1.62 mol/L glycerol including linear regression after the inflection point, point at which the reaction temperature of 115 °C was reached ($\dot{V}_g$ = 650 $\frac{nmL}{min}$,$\dot{V}_l$ = 1729 $\frac{mL}{min}$, 5 bar_{Oxygen}, 115 °C, 5 $\frac{mmol}{L}$ HPA-2, 6 h).

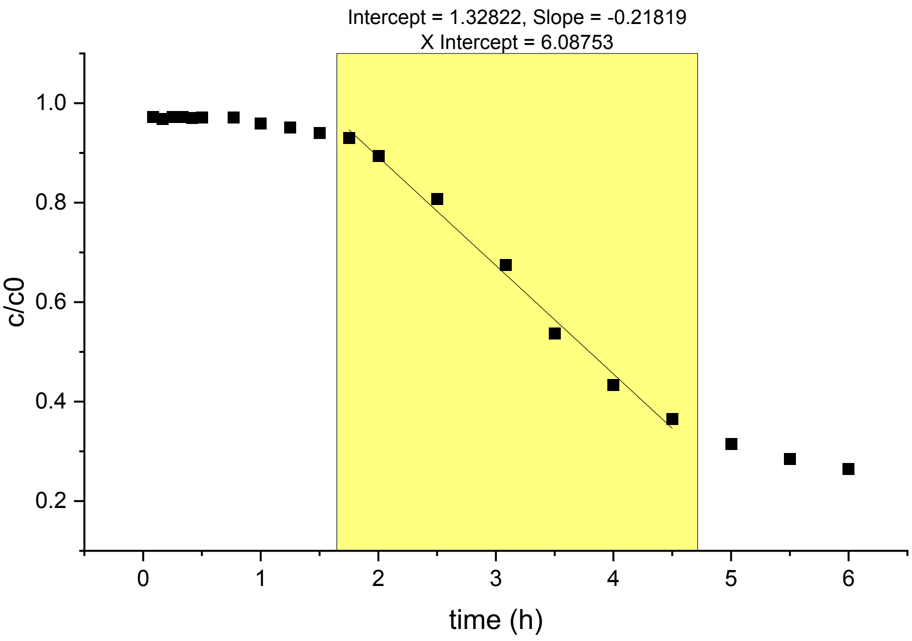

Figure S33: Normalized glycerol concentrations versus the reaction time of the experiment with 0.81 mol/L glycerol including linear regression after the inflection point, point at which the reaction temperature of 115 °C was reached ($\dot{V}_g$ = 650 $\frac{nmL}{min}$,$\dot{V}_l$ = 1729 $\frac{mL}{min}$, 5 bar_{Oxygen}, 115 °C, 5 $\frac{mmol}{L}$ HPA-2, 6 h).

10 Diagrams for determining the reaction order of oxygen

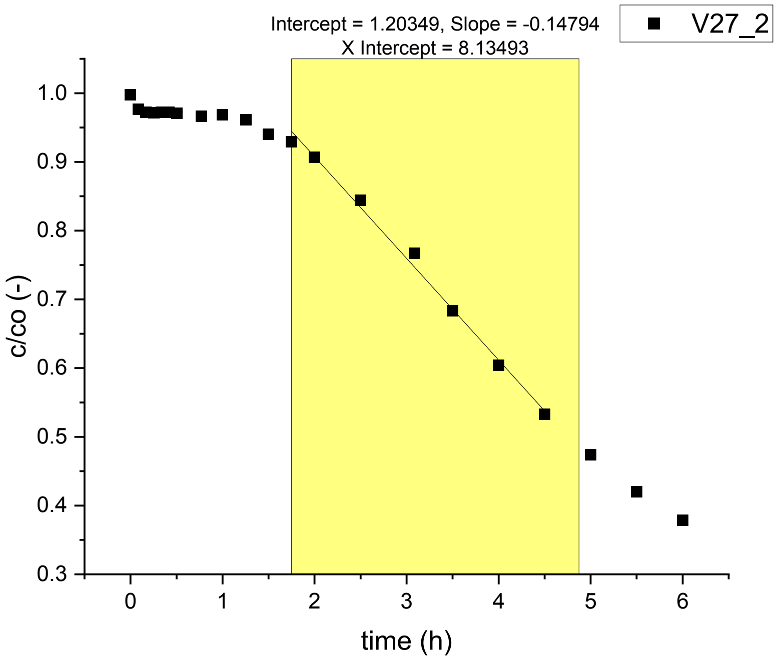

Figure S34: Normalized glycerol concentrations versus the reaction time of the experiment with 1.02 mol/L glycerol including linear regression after the inflection point, point at which the reaction temperature of 115 °C was reached ($\dot{V}_g$ = 650 $\frac{\text{nmL}}{\text{min}}$,$\dot{V}_l$ = 1729 $\frac{\text{mL}}{\text{min}}$, *$p_{O_2}$ = 1.96 bar*, 5 bar$_{\text{total}}$, 115 °C, 5 $\frac{\text{mmol}}{\text{L}}$ HPA-2, 6 h).

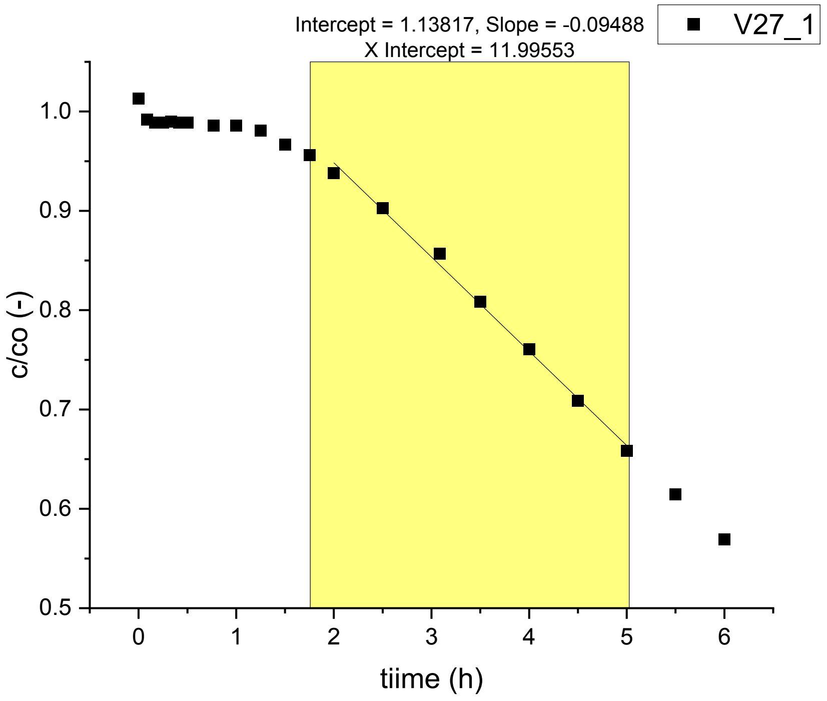

Figure S35: Normalized glycerol concentrations versus the reaction time of the experiment with 1.00 mol/L glycerol including linear regression after the inflection point, point at which the reaction temperature of 115 °C was reached ($\dot{V}_g$ = 650 $\frac{\text{nmL}}{\text{min}}$,$\dot{V}_l$ = 1729 $\frac{\text{mL}}{\text{min}}$, *air, p_{O_2} = 1.04 bar,* 5 bar$_{\text{total}}$, 115 °C, 5 $\frac{\text{mmol}}{\text{L}}$ HPA-2, 6 h).

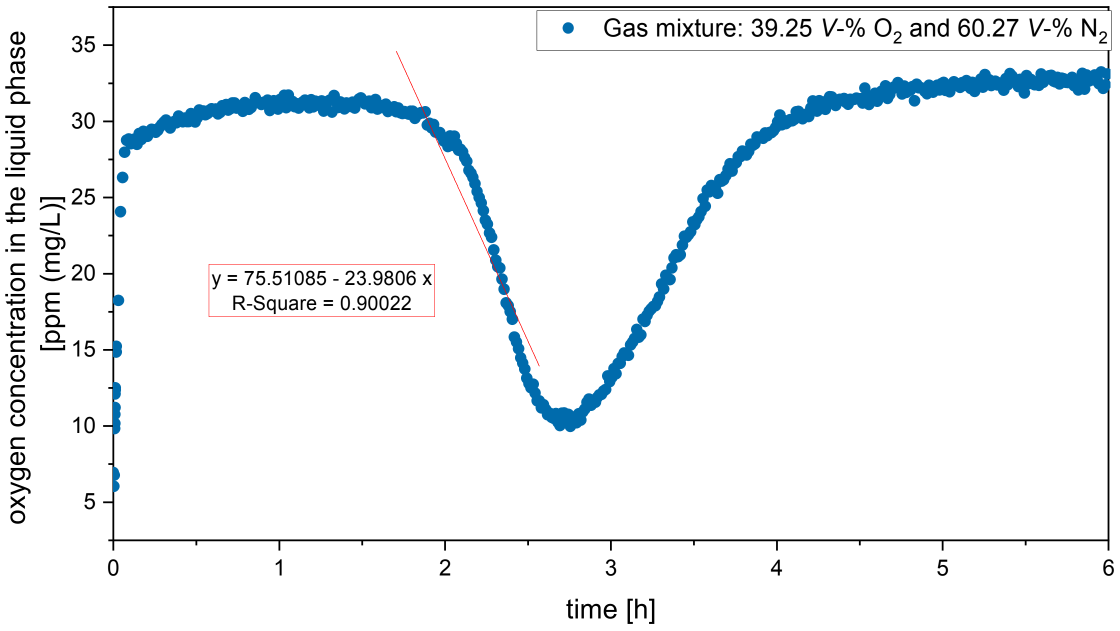

Figure S361: Oxygen concentration in the liquid phase versus the reaction time of the experiment with p_{Oxygen} = 1.96 bar including linear regression after the inflection point, point at which the reaction temperature of 115 °C was reached ($\dot{V}_g$ = 650 $\frac{\text{nmL}}{\text{min}}$,$\dot{V}_l$ = 1729 $\frac{\text{mL}}{\text{min}}$, p = 5 $\text{bar}_{\text{Total}}$, 115 °C, 5 $\frac{\text{mmol}}{\text{L}}$ HPA-2, 6 h).

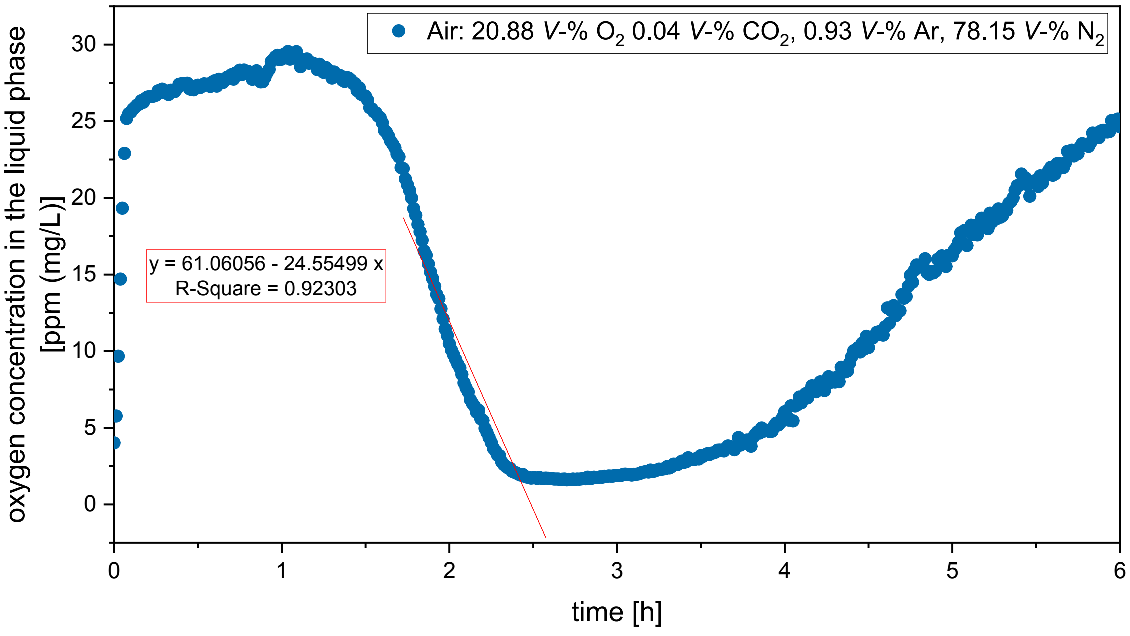

Figure S37*:* Oxygen concentration in the liquid phase versus the reaction time of the experiment with p_{Oxygen} = 1.04 bar including linear regression after the inflection point, point at which the reaction temperature of 115 °C was reached ($\dot{V}_g$ = 650 $\frac{\text{nmL}}{\text{min}}$,$\dot{V}_l$ = 1729 $\frac{\text{mL}}{\text{min}}$, p = 5 $\text{bar}_{\text{Total}}$, 115 °C, 5 $\frac{\text{mmol}}{\text{L}}$ HPA-2, 6 h).

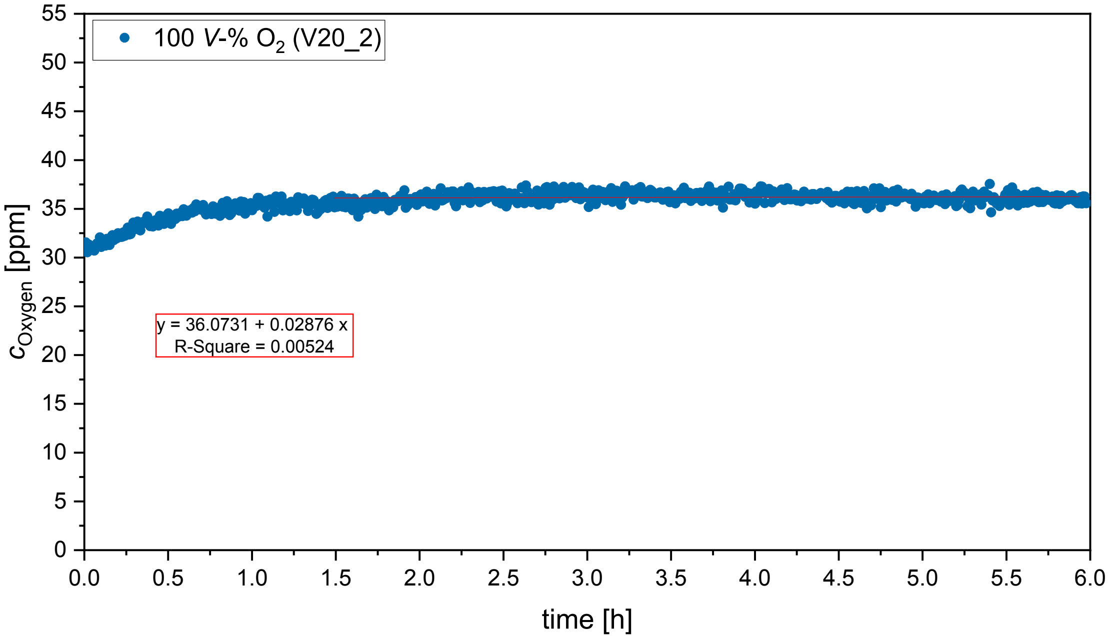

Figure S38: Concentration curve of oxygen in the liquid phase in the reference experiment with 1.06 mol/L glycerol plotted against reaction time ($\dot{V}_g$ = 650 $\frac{nmL}{min}$,$\dot{V}_l$ = 1729 $\frac{mL}{min}$, 5 bar_{Oxygen}, 115 °C, 10 *wt.*% glycerol, 5 $\frac{mmol}{L}$ HPA-2, 6 h).

11 Stability of the catalyst – interval charge transfer (IVCT)

UV-Vis spectra were recorded to analyze the redox state of the catalyst during the reaction. The measurement was carried with the undiluted reaction solution from 500 nm – 1000 nm for interval charge transfer. Each measurement was performed in duplicate on a CARY 60 UV-VIS spectrometer from AGILENT TECHNOLOGIES. The measurements were performed using the CARY WIN UV SCAN APPLICATION version 5.1.3.1042 software. The data were exported as a .csv file and plotted using ORIGIN software.

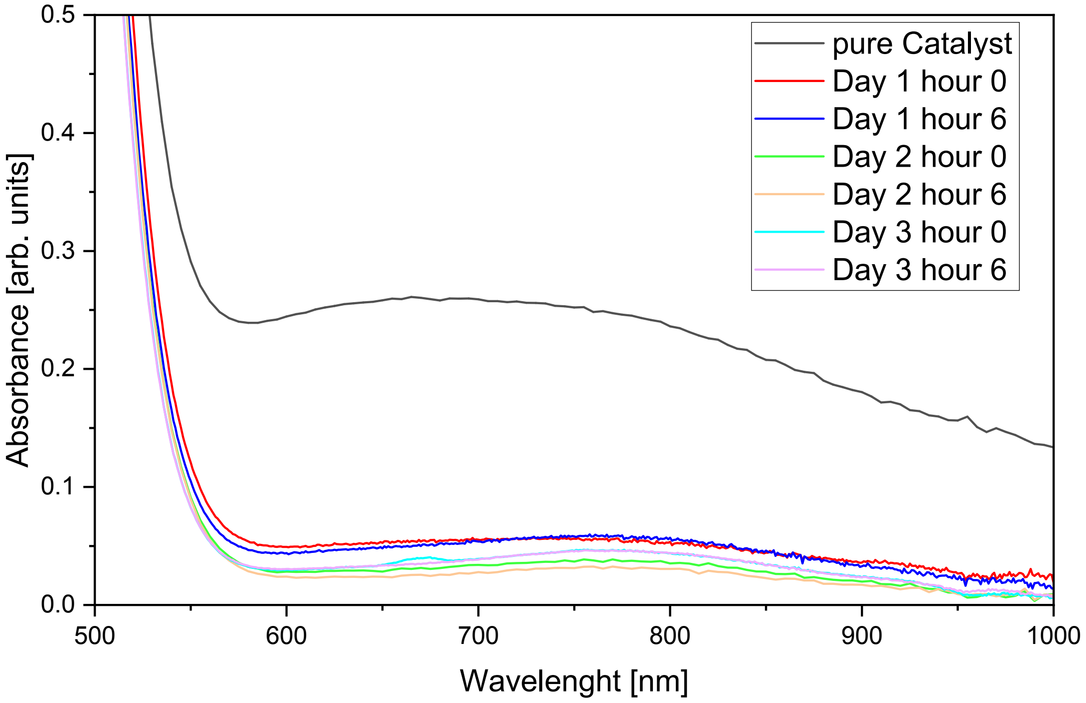

Figure S39: Absorbance of the reaction solution with homogeneous catalyst after different reaction times versus wavelength (400 – 1100 nm) in experiments with 1.01 mol/L glycerol including trend lines ($\dot{V}_g$ = 650 $\frac{nmL}{min}$,$\dot{V}_l$ = 1729 $\frac{mL}{min}$, 5 bar_{Oxygen}, 115 °C, 10 *wt.*% glycerol, 5 $\frac{mmol}{L}$ HPA-2, 6 h).

12 References

[1] V. F. Odyakov, E. G. Zhizhina, *Reaction Kinetics and Catalysis Letters* **2008**, *95*, 21-28.

[2] L. Pettersson, I. Andersson, J. H. Grate, A. Selling, *Inorganic Chemistry* **1994**, *33*, 982-993.

[3] J.-C. Raabe, J. Aceituno Cruz, J. Albert, M. J. Poller, *Inorganics* **2023**, *11*, 138.

[4] H. Landolt, R. Börnstein, K.-H. Hellwege, O. Madelung, *Numerical data and functional relationships in science and technologynew series*, Springer, Springer Berlin, **1961**.

[5] M. Maly, S. Schaper, R. Kuwertz, M. Hoffmann, J. Heck, M. Schlüter, *Processes* **2022**, *10*, 1531.
